# Current Awareness on Comparative and Functional Genomics

**DOI:** 10.1002/cfg.121

**Published:** 2002-12


**Comparative and Functional Genomics**
*Comp Funct Genom* 2002; **3:** 551–562
Published online in Wiley InterScience (www.interscience.wiley.com). **DOI** 10.1002/cfg.121

### Current awareness on comparative and functional genomics

CURRENT AWARENESS - USE THE LINKS ONLINE

View this bibliography online at WILEY InterScience www.interscience.wiley.com

Your gateway to Wiley journals online

The articles use crossref, the innovative multi-publisher reference linking. This enables readers to move seamlessly from the reference to the cited publication, typically located on a different server and published by a different publisher. Links to MEDLINE abstracts are also available for key journals in the biosciences.

2745

### 1 Reviews & symposia

2002. Special issue: Current problems of evolutionary genomics. *Russ J Genet* **38:** (6)

2002. Special issue: Genomics and the prospects of antibacterial & antifungal agents. *Curr Pharm Design* **8:** (13)

2002. Special issue: Proteomics databases Part I. *J Chromatogr B* **771:** (1-2)

2001. Special issue: Sugarcane transcriptome: A landmark in plant genomics in the tropics. *Genet Mol Biol* **24:** (1-4)

2002. Special issue on the *Arabidopsis* genome. *Plant Physiol* **129:** (2)

Afshari CA. 2002. NIEHS, Mol Carcinogenesis Lab, POB 12233, 111 TW Alexander Dr, Res Triangle Pk, NC 27709, USA. Perspective: Microarray technology, seeing more than spots. *Endocrinology* **143:** (6) 1983.

Auerbach D, Thaminy S, Hottiger MO, Stagljar I*. 2002. *Univ Zurich Irchel, Inst Vet Biochem & Mol Biol, Winterthurerstr 190, CH-8057 Zurich, Switzerland. The post-genomic era of interactive proteomics: Facts and perspectives - Review. *Proteomics* **2:** (6) 611.

Banerjee N, Zhang MX. 2002. CSH Lab, 1 Bungtown Rd, Cold Spring Harbor, NY 11724, USA. Functional genomics as applied to mapping transcription regulatory networks. *Curr Opin Microbiol* **5:** (3) 318.

Ben Shlomo I, Vitt UA, Hsueh AJW*. 2002. *Stanford Univ, Div Reprod Biol, 300 Pasteur Dr, Stanford, Ca 94305, USA. Perspective: The ovarian kaleidoscope database - II. Functional genomic analysis of an organ-specific database. *Endocrinology* **143:** (6) 2041.

Bevan M. 2002. John Innes Ctr, Dept Cell & Dev Biol, Colney Lane, Norwich NR4 7UJ, England. Genomics and plant cells: Application of genomics strategies to *Arabidopsis* cell biology. *Philos Trans R Soc Lond B* **357:** (1422) 731.

Bono H, Okazaki Y. 2002. RIKEN, Lab Genome Explorat Res Grp, Tsurumi ku, 1-7-22 Suehiro cho, Yokohama, Kanagawa 230 004, Japan. Functional transcriptomes: Comparative analysis of biological pathways and processes in eukaryotes to infer genetic networks among transcripts. *Curr Opin Struct Biol* **12:** (3) 355.

Burley SK, Bonanno JB. 2002. Structural GenomiX Inc, 10505 Roselle St, San Diego, Ca 92121, USA. Structural genomics of proteins from conserved biochemical pathways and processes. *Curr Opin Struct Biol* **12:** (3) 383.

Figeys D. 2002. MDS Proteomics, 251 Attwell Dr, Toronto, Ontario, Canada M9W 7H4. Adapting arrays and lab-on-a-chip technology for proteomics (Review). *Proteomics* **2:** (4) 373.

Foret F, Preisler J. 2002. Acad Sci Czech Republic, Inst Analyt Chem, Brno, Czech Republic. Liquid phase interfacing and miniaturization in matrix-assisted laser desorption/ionization mass spectrometry (Review). *Proteomics* **2:** (4) 360.

Gaucher EA, Gu X, Miyamoto MM, Benner SA*. 2002. *Univ Florida, NASA, Astrobiol Inst, Gainesville, Fl 32611, USA. Predicting functional divergence in protein evolution by site-specific rate shifts (Review). *Trends Biochem Sci* **27:** (6) 315.

Gorski S, Marra M. 2002. BC Canc Agcy, Genome Sequence Ctr, 600 West 10th Ave, Vancouver, Brit Columbia, Canada V5Z 4E5. Programmed cell death takes flight: Genetic and genomic approaches to gene discovery in *Drosophila* (Review). *Physiol Genomics* **9:** (2) 59.

Hancock WS, Wu SL, Shieh P. 2002. Thermo Finnigan, 255 River Oaks Pkwy, San Jose, Ca 95134, USA. The challenges of developing a sound proteomics strategy (Review). *Proteomics* **2:** (4) 352.

Harrison PM, Gerstein M*. 2002. *Yale Univ, Dept Mol Biophys & Biochem, 266 Whitney Ave, New Haven, Ct 06520, USA. Studying genomes through the aeons: Protein families, pseudogenes and proteome evolution (Review). *J Mol Biol* **318:** (5) 1155.

Hockett RD, Kirkwood SC, Mitlak BH, Dere WH. 2002. Eli Lilly & Co, Lilly Corp Ctr, Indianapolis, In 46285, USA. Perspective: Pharmacogenomics in endocrinology. *J Clin Endocrinol Metab* **87:** (6) 2495.

Iida K, Nishimura I*. 2002. *UCLA, Sch Dent, Weintraub Ctr Reconstruct Biotechnol, Los Angeles, Ca 90095, USA. Gene expression profiling by DNA microarray technology. *Crit Rev Oral Biol Med* **13:** (1) 35.

Kodadek T. 2002. UT SW Med Ctr, Dept Internal Med, 5323 Harry Hines Blvd, Dallas, Tx 75390, USA. Development of protein-detecting microarrays and related devices (Review). *Trends Biochem Sci* **27:** (6) 295.

Kopchick JJ, List EO, Kohn DT, Keidan GMO, Qiu L, Okada S. 2002. Ohio Univ, Edison Biotechnol Inst, Konneker Res Labs, Athens, Oh 45701, USA. Perspective: Proteomics - See "spots" run. *Endocrinology* **143:** (6) 1990.

In order to keep subscribers up-to-date with the latest developments in their field, this current awareness service is provided by John Wiley & Sons and contains newly-published material on comparative and functional genomics. Each bibliography is divided into 16 sections. 1 Reviews & symposia; 2 General; 3 Large-scale sequencing and mapping; 4 Evolutionary genomics; 5 Comparative genomics; 6 Pathways, gene families and regulons; 7 Pharmacogenomics; 8 EST, cDNA and other clone resources; 9 Functional genomics; 10 Transcriptomics; 11 Proteomics; 12 Protein structural genomics; 13 Metabolomics; 14 Genomic approaches to development; 15 Technological advances; 16 Bioinformatics. Within each section, articles are listed in alphabetical order with respect to author. If, in the preceding period, no publications are located relevant to any one of these headings, that section will be omitted.

Copyright © 2002 John Wiley & Sons, Ltd.

Laurell T, Marko-Varga G*. 2002. *Lund Univ, Dept Analyt Chem, Box 124, SE-22100 Lund, Sweden. Miniaturisation is mandatory unravelling the human proteome (Review). *Proteomics* **2:** (4) 345.

Leo CP, Hsu SY, Hsueh AJW*. 2002. *Stanford Univ, Div Reprod Biol, 300 Pasteur Dr, Stanford, Ca 94305, USA. Hormonal genomics. *Endocr Rev* **23:** (3) 369.

Maniatis T, Tasic B. 2002. Harvard Univ, Dept Cellular & Mol Biol, Cambridge, Ma 02138, USA. Alternative pre-mRNA splicing and proteome expansion in metazoans (Review). *Nature* **418:** (6894) 236.

Mann M, Ong SE, Gronborg M, Steen H, Jensen ON, Pandey A. 2002. Univ Sthn Denmark, Ctr Expt Bioinformatics, DK-5230 Odense M, Denmark. Analysis of protein phosphorylation using mass spectrometry: Deciphering the phosphoproteome (Review). *Trends Biotechnol* **20:** (6) 261.

Medh RD. 2002. Calif State Univ, Dept Biol, 18111 Nordhoff St, Northridge, Ca 91330, USA. Microarray-based expression profiling of normal and malignant immune cells. *Endocr Rev* **23:** (3) 393.

Meidanis J, Braga MDV, Verjovski-Almeida S*. 2002. *Univ Sao Paulo, Inst Quim, Avda Prof Lineu Prestes 748, BR-05508-900 Sao Paulo, Brazil. Whole-genome analysis of transporters in the plant pathogen *Xylella fastidiosa. Microbiol Mol Biol Rev* **66:** (2) 272.

Mong JA, Krebs C, Pfaff DW. 2002. Rockefeller Univ, Neurobiol & Behav Lab, 1230 York Ave, New York, NY 10021, USA. Perspective: Microarrays and differential display PCR - Tools for studying transcript levels of genes in neuroendocrine systems. *Endocrinology* **143:** (6) 2002.

Mukherjee D, Topol EJ*. 2002. *Cleveland Clin Fdn, Dept Cardiovasc Med, 9500 Euclid Ave, Cleveland, Oh 44195, USA. Pharmacogenomics in cardiovascular diseases. *Prog Cardiovasc Dis* **44:** (6) 479.

Nadon R, Shoemaker J. 2002. Brock Univ, Imaging Res Inc, 500 Glenridge Ave, St Catharines, Ontario, Canada L2S 3A1. Statistical issues with microarrays: Processing and analysis. *Trends Genet* **18:** (5) 265.

Olson LE, Rosenfeld MG*. 2002. *Univ Calif San Diego, Eukaryot Reg Bio Program, 9500 Gilman Dr, La Jolla, Ca 92093, USA. Perspective: Genetic and genomic approaches in elucidating mechanisms of pituitary development. *Endocrinology* **143:** (6) 2007.

Pan W. 2002. Univ Minnesota, Div Biostat, 420 Delaware St SE, Minneapolis, Mn 55455, USA. A comparative review of statistical methods for discovering differentially expressed genes in replicated microarray experiments. *Bioinformatics* **18:** (4) 546.

Rust AG, Mongin E, Birney E. 2002. EMBL, EBI, Wellcome Trust Genome Campus, Cambridge CB10 1SD, England. Genome annotation techniques: New approaches and challenges. *Drug Discov Today* **7:** (11) S70.

Sanford K, Soucaille P, Whited G, Chotani G. 2002. Genencor Int Inc, 925 Page Mill Rd, Palo Alto, Ca 94304, USA. Genomics to fluxomics and physiomics: Pathway engineering. *Curr Opin Microbiol* **5:** (3) 318.

Shoemaker DD, Linsley PS. 2002. Rosetta Inpharmatics, 12040 115th Ave NE, Kirkland, Wa 98034, USA. Recent developments in DNA microarrays. *Curr Opin Microbiol* **5:** (3) 334.

Soanes DM, Skinner W, Keon J, Hargreaves J, Talbot NJ*. 2002. *Univ Exeter, Washington Singer Labs, Perry Rd, Exeter EX4 4QG, England. Genomics of phytopathogenic fungi and the development of bioinformatic resources (Review). *Mol Plant Microbe Interact* **15:** (5) 421.

Soulet D, Rivest S. 2002. CHU Laval, Mol Endocrinol Lab, 2705 Blvd Laurier, Quebec City, Quebec, Canada G1V 4G2. Perspective: How to make microarray, serial analysis of gene expression, and proteomic relevant to day-to-day endocrine problems and physiological systems. *Endocrinology* **143:** (6) 1995.

Stormo GD, Tan K. 2002. Washington Univ, Dept Genet, St Louis, Md 63110, USA. Mining genome databases to identify and understand new gene regulatory systems. *Curr Opin Microbiol* **5:** (2) 149.

Wood V, Bahler J*. 2002. *Wellcome Trust Sanger Inst, Hinxton, Cambridge CB10 1SA, England. Website Review: How to get the best from fission yeast genome data. *Comp Funct Genom* **3:** (3) 282.

Xing T, Ouellet T, Miki BL. 2002. Agr & Agri-Food Canada, Cereal Res Ctr, Winnipeg, Manitoba, Canada R3T 2M9. Towards genomic and proteomic studies of protein phosphorylation in plant-pathogen interactions (Review). *Trends Plant Sci* **7:** (5) 224.

### 3 Large-scale sequencing and mapping

Bao QY, Tian YQ, Li W, Xu ZY, Xuan ZY, Hu SN, Dong W, Yang J, Chen YJ, Xue YF *et al*. 2002. c/o Tan HR, Chinese Acad Sci, Inst Microbiol, CN-100080 Beijing, Peoples Rep China. A complete sequence of the *T. tengcongensis* genome. *Genome Res* **12:** (5) 689.

Ogata H, Audic S, Abergel C, Fournier PE, Claverie JM. 2002. CNRS-AVENTIS UMR 1889, FR-13402 Marseille 20, France. Protein coding palindromes are a unique but recurrent feature in *Rickettsia*. *Genome Res* **12:** (5) 808.

### 4 Evolutionary genomics

Bennetzen JL. 2002. Purdue Univ, Dept Biol Sci, West Lafayette, In 47907, USA. Mechanisms and rates of genome expansion and contraction in flowering plants. *Genetica* **115:** (1) 29.

Betran E, Long MY. 2002. Univ Chicago, Dept Ecol & Evolut, 304 Zool Bldg, 1101 East 57th St, Chicago, Il 60637, USA. Expansion of genome coding regions by acquisition of new genes. *Genetica* **115:** (1) 65.

Birdsell JA. 2002. Univ Arizona, Dept Ecol & Evolutionary Biol, Tucson, Az 85121, USA. Integrating genomics, bioinformatics, and classical genetics to study the effects of recombination on genome evolution. *Mol Biol Evol* **19:** (7) 1181.

Castillo-Davis CI, Hartl DL*. 2002. *Harvard Univ, Dept Organism & Evolutionary Biol, 16 Divin Ave, Cambridge, Ma 02138, USA. Genome evolution and developmental constraint in *Caenorhabditis elegans*. *Mol Biol Evol* **19:** (5) 728.

Fahey ME, Moore TF, Higgins DG*. 2002. *Univ Coll Cork, Dept Biochem, Lee Maltings, Prospect Row, Cork, Rep Ireland. Overlapping antisense transcription in the human genome. *Comp Funct Genom* **3:** (3) 244.

Frank AC, Amiri H, Andersson SGE*. 2002. *Univ Uppsala, Dept Mol Evolut, SE-75136 Uppsala, Sweden. Genome deterioration: Loss of repeated sequences and accumulation of junk DNA. *Genetica* **115:** (1) 1.

Gegory TR. 2002. Univ Guelph, Dept Zool, Guelph, Ontario, Canada N1G 2W1. Genome size and developmental complexity. *Genetica* **115:** (1) 131.

Gilson PR, McFadden GI*. 2002. *Univ Melbourne, Sch Bot, Plant Cell Biol Res Ctr, Parkville, Vic 3010, Australia. Jam packed genomes: A preliminary, comparative analysis of nucleomorphs. *Genetica* **115:** (1) 13.

Hancock JM. 2002. Univ London, Royal Holloway & Bedford New Coll, Dept Comp Sci, Egham TW20 0EX, England. Genome size and the accumulation of simple sequence repeats: Implications of new data from genome sequencing projects. *Genetica* **115:** (1) 93.

Kidwell MG. 2002. Univ Arizona, Dept Ecol & Evolutionary Biol, Tucson, Az 85721, USA. Transposable elements and the evolution of genome size in eukaryotes. *Genetica* **115:** (1) 49.

Liu GR, Rahn A, Liu WQ, Sanderson KE, Johnston RN, Liu SL*. 2002. *Univ Calgary, Dept Microbiol & Infect Dis, 3330 Hosp Dr NW, Calgary, Alberta, Canada T2N 4N1. The evolving genome of *Salmonella enterica* serovar Pullorum. *J Bacteriol* **184:** (10) 2626.

McLysaght A, Hokamp K, Wolfe KH*. 2002. *Univ Dublin, Trinity Coll, Dept Genet, Smurfit Inst, Dublin 2, Rep Ireland. Extensive genomic duplication during early chordate evolution. *Nat Genet* **31:** (2) 200.

Naimuddin M, Kurazono T, Nishigaki K*. 2002. *Saitama Univ, Dept Funct Mat Sci, 255 Shimo Okubo, Saitama 338 8570, Japan. Commonly conserved genetic fragments revealed by genome profiling can serve as tracers of evolution (Online paper). *Nucleic Acids Res* **30:** (10) E42.

Petrov DA. 2002. Stanford Univ, Dept Biol Sci, 371 Serra St, Stanford, Ca 94305, USA. DNA loss and evolution of genome size in *Drosophila*. *Genetica* **115:** (1) 81.

Porwollik S, Wong RMY, McClelland M*. 2002. *Sidney Kimmel Canc Ctr, 10835 Altman Row, San Diego, Ca 92121, USA. Evolutionary genomics of *Salmonella*: Gene acquisitions revealed by microarray analysis. *Proc Natl Acad Sci U S A* **99:** (13) 8956.

Smith NGC, Eyre-Walker A. 2002. Uppsala Univ, Dept Evolutionary

Copyright © 2002 John Wiley & Sons, Ltd.

Biol, Evolutionary Biol Ctr, Norbyvagen 18D, SE-75236 Uppsala, Sweden. The compositional evolution of the murid genome. *J Mol Evol* **55:** (2) 197.

Wendel JF, Cronn RC, Johnston JS, Price HJ. 2002. Iowa State Univ, Dept Bot, Ames, Ia 50011, USA. Feast and famine in plant genomes. *Genetica* **115:** (1) 37.

Zuckerkandl E. 2002. Inst Mol Sci Med, POB 20452, Stanford, Ca 94309, USA. Why so many noncoding nucleotides? The eukaryote genome as an epigenetic machine. *Genetica* **115:** (1) 105.

### 5 Comparative genomics

Agron PG, Macht M, Radnedge L, Skowronski EW, Miller W, Andersen GL*. 2002. *Lawrence Livermore Natl Lab, Biol & Biotechnol Res Program, 7000 East Ave, Livermore, Ca 94551, USA. Use of subtractive hybridization for comprehensive surveys of prokaryotic genome differences. *FEMS Microbiol Lett* **211:** (2) 175.

Cornillot E, Metenier G, Vivares CP*, Dassa E. 2002. *Univ Clermont Ferrand, UMR CNRS 6023, LBP, FR-63177 Clermont Ferrand, France. Comparative analysis of sequences encoding ABC systems in the genome of the microsporidian *Encephalitozoon cuniculi. FEMS Microbiol Lett* **210:** (1) 39.

Da Silva ACR, Ferro JA, Reinach FC, Farah CS, Furlan LR, Quaggio RB, Monteiro-Vitorello CB, Van Sluys MA, Almeida NF, Alves LMC *et al*. 2002. USP, Dept Bioquim, Avda Prof Lineu Prestes 748, BR-05508-900 Sao Paulo, Brazil. Comparison of the genomes of two *Xanthomonas* pathogens with differing host specifities. *Nature* **417:** (6887) 459.

Ebersberger I, Metzler D, Schwarz C, Paabo S*. 2002. *Max Planck Inst Evolut Anthropol, Inselstr 22, DE-04103 Leipzig, Germany. Genomewide comparison of DNA sequences between humans and chimpanzees. *Am J Hum Genet* **70:** (6) 1490.

Lapidus A, Galleron N, Andersen JT, Jorgensen PL, Ehrlich SD, Sorokin A*. 2002. *INRA, Domaine Vilvert, FR-78352 Jouy-en-Josas, France. Co-linear scaffold of the *Bacillus licheniformis* and *Bacillus subtilis* genomes and its use to compare their competence genes. *FEMS Microbiol Lett* **209:** (1) 23.

Muller F, Blader P, Strahle U. 2002. Inst Toxicol & Genet, Forschungszentrum, POB 3640, DE-76021 Karlsruhe, Germany. Search for enhancers: Teleost models in comparative genomic and transgenic analysis of *cis* regulatory elements. *Bioessays* **24:** (6) 564.

Mural RJ, Adams MD, Myers EW, Smith HO, Miklos GLG, Wides R, Halpern A, Li PW, Sutton GG, Nadeau J *et al*. 2002. Celera Genomics, 45 West Gude Dr, Rockville, Md 20850, USA. A comparison of whole-genome shotgun-derived mouse chromosome 16 and the human genome. *Science* **296:** (5573) 1661.

Read TD, Salzberg SL, Pop M, Shumway M, Umayam L, Jiang LX, Holtzapple E, Busch JD, Smith KL, Schupp JM *et al*. 2002. c/o Fraser CM, Inst Genomic Res, 9712 Ctr Dr, Rockville, Md 20850, USA. Comparative genome sequencing for discovery of novel polymorphisms in *Bacillus anthracis*. *Science* **296:** (5575) 2028.

Salse J, Piegu B, Cooke R*, Delseny M. 2002. *Univ Perpignan, CNRS, UMR 5096, LGDP, 52 Ave Valleneuve, FR-66860 Perpignan, France. Synteny between *Arabidopsis thaliana* and rice at the genome level: A tool to identify conservation in the ongoing rice genome sequencing project. *Nucleic Acids Res* **30:** (11) 2316.

Thomasova D, Ton LQ, Copley RR, Zdobnov EM, Wang XL, Hong YS, Sim C, Bork P, Kafatos FC*, Collins FH. 2002. *EMBL, Meyerhofstr 1, DE-69117 Heidelberg, Germany. Comparative genomic analysis in the region of a major *Plasmodium*-refractoriness locus of *Anopheles gambiae*. *Proc Natl Acad Sci U S A* **99:** (12) 8179.

### 6 Pathways, gene families and regulons

Bowles D. 2002. Univ York, Dept Biol, Ctr Novel Agr Prod, York YO10 5YW, England. A multigene family of glycosyltransferases in a model plant, *Arabidopsis thaliana*. *Biochem Soc Trans* **30:** (Apr) 301.

Hussey PJ, Allwood EG, Smertenko AP. 2002. Univ Durham, Sch Biol & Biomed Sci, Integrat Cell Biol Lab, South Rd, Durham DH1 3LE, England. Actin-binding proteins in the *Arabidopsis* genome database: Properties of functionally distinct plant actin-depolymerizing factors/cofilins. *Philos Trans R Soc Lond B* **357:** (1422) 791.

Van Nimwegen E, Zavolan M, Rajewsky N, Siggia ED. 2002. Rockefeller Univ, Ctr Studies Phys & Biol, 1230 York Ave, New York, NY 10021, USA. Probabilistic clustering of sequences: Inferring new bacterial regulons by comparative genomics. *Proc Natl Acad Sci U S A* **99:** (11) 7323.

### 7 Pharmacogenomics

Alaiya AA, Franzen B, Hagman A, Dysvik B, Roblick UJ, Becker S, Moberger B, Auer G, Linder S. 2002. Karolinska Hosp, Dept Pathol & Oncol, Canc Ctr, SE-17176 Stockholm, Sweden. Molecular classification of borderline ovarian tumors using hierarchical cluster analysis of protein expression profiles. *Int J Cancer* **98:** (6) 895.

Anderson KM, Alrefai WA, Anderson CA, Ho Y, Jadko S, Ou D, Wu YB, Harris JE. 2002. Rush Med Coll, Dept Med, 1753 West Harrison, Chicago, Il 60612, USA. A response of Panc-1 cells to *cis*-platinum, assessed with a cDNA array. *Anticancer Res* **22:** (1A) 75.

Anzick SL, Trent JM*. 2002. *NIH/Natl Human Genome Res Inst, Canc Genet Branch, 50 Sth Dr, Bethesda, Md 20892, USA. Role of genomics in identifying new targets for cancer therapy. *Oncology - NY* **16:** (5 Suppl 4) 7.

Asmann YW, Kosari F, Wang K, Cheville JC, Vasmatzis G*. 2002. *Mayo Clin & Fdn, Dept Lab Med & Pathol, 200 1st St SW, Rochester, Mn 55905, USA. Identification of differentially expressed genes in normal and malignant prostate by electronic profiling of expressed sequence tags. *Cancer Res* **62:** (11) 3308.

Bangur CS, Switzer A, Fan LQ, Marton MJ, Meyer MR, Wang TT. 2002. Corixa Corp, Tumor Antigen Discovery, 1124 Columbia St, Seattle, Wa 98104, USA. Identification of genes over-expressed in small cell lung carcinoma using suppression subtractive hybridization and cDNA microarray expression analysis. *Oncogene* **21:** (23) 3814.

Barrans JD, Allen PD, Stamatiou D, Dzau VJ, Liew CC*. 2002. *Harvard Univ, Brigham & Womens Hosp, Cardiovasc Genome Unit, 75 Francis St, Boston, Ma 02115, USA. Global gene expression profiling of end-stage dilated cardiomyopathy using a human cardiovascular-based cDNA microarray. *Am J Pathol* **160:** (6) 2035.

Bell PA, Chaturvedi S, Gelfand CA, Huang CY, Kochersperger M, Kopla R, Modica F, Pohl M, Varde S, Zhao RB *et al*. 2002. c/o Boyce-Jacino MT, Orchid Biosciences, 303 Coll Rd East, Princeton, NJ 08540, USA. SNPstream® UHT: Ultra-high throughput SNP genotyping for pharmacogenomics and drug discovery. *Biotechniques* **32:** (Suppl) 70.

Bertucci F, Nasser V, Granjeaud S, Eisinger F, Adelaide J, Tagett R, Loriod A, Giaconia A, Benziane A, Devilard E *et al*. 2002. c/o Birnbaum D, IFR 57, U119 INSERM, Lab Oncol Mol, 27 Blvd Lei Roure, FR-13009 Marseille, France. Gene expression profiles of poor-prognosis primary breast cancer correlate with survival. *Hum Mol Genet* **11:** (8) 863.

Cadet JL, McCoy MT, Ladenheim B. 2002. NIH/NIA, Mol Neuropsychiat Sect, POB 5160, Baltimore, MD 21224, USA. Distinct gene expression signatures in the striata of wild-type and heterozygous c-fos knockout mice following methamphetamine administration: Evidence from cDNA array analyses. *Synapse* **44:** (4) 211.

Crnogorac-Jurcevic T, Efthimiou E, Nielsen T, Loader J, Terris B, Stamp G, Baron A, Scarpa A, Lemoine NR*. 2002. *Univ London, Imperial Coll Sci Technol & Med, Sch Med, Dept Canc Med, Mol Oncol Unit, London W12 0NN, England. Expression profiling of microdissected pancreatic adenocarcinomas. *Oncogene* **21:** (29) 4587.

Dangles V, Lazar V, Validire P, Richon S, Wertheimer M, Laville V, Janneau JL, Barrois M, Bovin C, Poynard T *et al*. 2002. c/o Bellet D, Inst Gustave Roussy, Dept Biol Clin, rue Camille Desmoulins, FR-94805 Villejuif, France. Gene expression profiles of bladder cancers: Evidence for a striking effect of *in vitro* cell models on gene patterns. *Br J Cancer* **86:** (8) 1283.

De la Vega FM, Dailey D, Ziegle J, Williams J, Madden D, Gilbert DA. 2002. Appl Biosyst Inc, 850 Lincoln Ctr Dr, Foster City, Ca 94404, USA. New generation pharmacogenomic tools: A SNP linkage disequilibrium map, validated SNP assay resource, and high-throughput instrumentation system for large-scale genetic studies. *Biotechniques*

Copyright © 2002 John Wiley & Sons, Ltd. 

**32:** (Suppl) 48.

Devilard E, Bertucci F, Trempat P, Bouabdallah R, Loriod B, Giaconia A, Brousset P, Granjeaud S, Nguyen C, Birnbaum D *et al*. 2002. c/o Xerri L, 232 Blvd St Marguerite, BP 156, FR-13273 Marseille 9, France. Gene expression profiling defines molecular subtypes of classical Hodgkin's disease. *Oncogene* **21:** (19) 3095.

Duckworth DM, Sanseau P. 2002. GlaxoSmithKline, Discovery Bioinformat Europe, Gunnels Wood Rd, Stevenage SG1 2NY, England. *In silico* identification of novel therapeutic targets. *Drug Discov Today* **7:** (11) S64.

Ellis M, Davis N, Coop A, Liu M, Schumaker L, Lee RY, Srikanchana R, Russell CG, Singh B, Miller WR *et al*. 2002. c/o Clarke R, Georgetown Univ, Dept Oncol, 3970 Reservoir Rd NW, Washington, DC 20007, USA. Development and validation of a method for using breast core needle biopsies for gene expression microarray analyses. *Clin Cancer Res* **8:** (5) 1155.

Fisher MA, Plikaytis BB, Shinnick TM*. 2002. *Ctr Dis Control & Prevent, Mailstop G35, Atlanta, Ga 30333, USA. Microarray analysis of the *Mycobacterium tuberculosis* transcriptional response to the acidic conditions found in phagosomes. *J Bacteriol* **184:** (14) 4025.

Fujii T, Dracheva T, Player A, Chacko S, Clifford R, Strausberg RL, Buetow K, Azumi N, Travis WD, Jen J*. 2002. *NIH/NCI, Lab Populat Genet, Ctr Canc Res, 41 Liberty Dr, Bldg 41 Room D702, Bethesda, Md 20892, USA. A preliminary transcriptome map of non-small cell lung cancer. *Cancer Res* **62:** (12) 3340.

Hamadeh HK, Bushel PR, Jayadev S, Martin K, DiSorbo O, Sieber S, Bennett L, Tennant R, Stoll R, Barrett JC *et al*. 2002. c/o Afshari CA, NIEHS, POB 12233, MD2-04, Research Triangle Park, NC 27709, USA. Gene expression analysis reveals chemical-specific profiles. *Toxicol Sci* **67:** (2) 219.

Hamadeh HK, Bushel PR, Jayadev S, DiSorbo O, Bennett L, Li LP, Tennant R, Stoll R, Barrett JC, Paules RS *et al*. 2002. c/o Afshari CA, Address as above. Prediction of compound signature using high density gene expression profiling. *Toxicol Sci* **67:** (2) 232.

Han HY, Bearss DJ, Browne LW, Calaluce R, Nagle RB, Von Hoff DD*. 2002. *Univ Arizona, Arizona Canc Ctr, POB 245024, Tucson, Az 85724, USA. Identification of differentially expressed genes in pancreatic cancer cells using cDNA microarray. *Cancer Res* **62:** (10) 2890.

Haviv I, Campbell IG*. 2002. *Peter MacCallum Canc Inst, VBCRC Canc Genet Lab, St Andrews Pl, Melbourne, Vic 3002, Australia. DNA microarrays for assessing ovarian cancer gene expression. *Mol Cell Endocrinol* **191:** (1) 121.

Koike E, Hirano S, Shimojo N, Kobayashi T. 2002. Natl Inst Environm Studies, Environm Hlth Sci Div, 16-2 Onogawa, Tsukuba, Ibaraki 305 8506, Japan. cDNA microarray analysis of gene expression in rat alveolar macrophages in response to organic extract of diesel exhaust particles. *Toxicol Sci* **67:** (2) 241.

Lee JS, Thorgeirsson SS*. 2002. *NIH/NCI, Canc Res Ctr, 37 Convent Dr, MSC 4258, Bldg 37, Rm 3C28, Bethesda, Md 20892, USA. Functional and genomic implications of global gene expression profiles in cell lines from human hepatocellular cancer. *Hepatology* **35:** (5) 1134.

Li HY, Jie SH, Zou P, Zou GL. 2002. Huazhong Univ Sci & Technol, Union Hosp, Tongji Med Coll, Jiefang Rd, CN-430022 Wuhan, Peoples Rep China. cDNA microarray analysis of chronic myeloid leukemia. *Int J Hematol* **75:** (4) 388.

Lock C, Hermans G, Pedotti R, Brendolan A, Schadt E, Garren H, Langer-Gould A, Strober S, Cannella B, Allard J *et al*. 2002. c/o Heller R, Roche Biosci, Palo Alto, Ca 94304, USA. Gene-microarray analysis of multiple sclerosis lesions yields new targets validated in autoimmune encephalomyelitis. *Nat Med* **8:** (5) 500.

Luo J, Dunn T, Ewing C, Sauvageot J, Chen YD, Trent J, Isaacs W*. 2002. *Johns Hopkins Med Inst, Dept Urol, 600 Nth Wolfe St, Baltimore, Md 21287, USA. Gene expression signature of benign prostatic hyperplasia revealed by cDNA microarray analysis. *Prostate* **51:** (3) 189.

Mellick AS, Day CJ, Weinstein SR, Griffiths LR, Morrison NA*. 2002. *Griffith Univ, Genomics Res Ctr, Gold Coast Mail Ctr PMB 50, Gold Coast Mail Ctr, Qld 9726, Australia. Differential gene expression in breast cancer cell lines and stroma-tumor differences in microdissected breast cancer biopsies revealed by display array analysis. *Int J Cancer* **100:** (2) 172.

Midorikawa Y, Tsutsumi S, Taniguchi H, Ishii M, Kobune Y, Kodama T, Makuuchi M, Aburatani H*. 2002. *Univ Tokyo, Genome Sci Div, Meguro ku, 4-6-1 Komaba, Tokyo 153 8904, Japan. Identification of genes associated with dedifferentiation of hepatocellular carcinoma with expression profiling analysis. *Jpn J Cancer Res* **93:** (6) 636.

Miura K, Bowman ED, Simon R, Peng AC, Robles AI, Jones RT, Katagiri T, He P, Mizukami H, Charboneau L *et al*. 2002. c/o Harris CC, NIH/NCI, Human Carcinogenesis Lab, 37 Convent Dr, Bethesda, Md 20892, USA. Laser capture microdissection and microarray expression analysis of lung adenocarcinoma reveals tobacco smoking- and prognosis-related molecular profiles. *Cancer Res* **62:** (11) 3244.

Moller A, Malerczyk C, Volker U, Stoppler H, Maser E*. 2002. *Univ Marburg, Sch Med, Dept Pharmacol & Toxicol, Karl von Frisch Str 1, DE-35033 Marburg, Germany. Monitoring daunorubicin-induced alterations in protein expression in pancreas carcinoma cells by two-dimensional gel electrophoresis. *Proteomics* **2:** (6) 697.

Narita N, Noda I, Ohtsubo T, Fujieda S, Tokuriki M, Saito T, Saito H. 2002. Fukui Med Univ, Dept Otorhinolaryngol, Fukui 910 1193, Japan. Analysis of heat-shock related gene expression in head-and-neck cancer using cDNA arrays. *Int J Radiat Oncol Biol Phys* **53:** (1) 190.

Nesic O, Svrakic NM, Xu GY, McAdoo D, Westlund KN, Hulsebosch CE, Ye ZM, Galante A, Soteropoulos P, Tolias P *et al*. 2002. UT Med Branch, Dept Human Biol Chem & Genet, 301 Univ Blvd, Galveston, Tx 77555, USA. DNA microarray analysis of the contused spinal cord: Effect of NMDA receptor inhibition. *J Neurosci Res* **68:** (4) 406.

Ohira T, Akutagawa S, Usuda J, Nakamura T, Hirano T, Tsuboi M, Nishio K*, Taguchi F, Ikeda N, Nakamura H *et al*. 2002. *Natl Canc Ctr, Div Pharmacol, Chuo ku, Tsukui 5-1-1, Tokyo 104 0045, Japan. Up-regulated gene expression of angiogenesis factors in post-chemotherapeutic lung cancer tissues determined by cDNA macroarray. *Oncol Rep* **9:** (4) 723.

Okuda T, Sumiya T, Muzutani K, Tago N, Miyata T, Tanabe T, Kato H, Katsuya T, Higaki J, Ogihara T *et al*. 2002. c/o Iwai N, Natl Cardiovascular Ctr, 5-7-1 Fujishirodai, Suita, Osaka 565 8565, Japan. Analyses of differential gene expression in genetic hypertensive rats by microarray. *Hypertens Res* **25:** (2) 249.

Owens GE, Keri RA, Nilson JH*. 2002. *Case Western Reserve Univ, Dept Pharmacol, 2109 Adelbert Rd, Cleveland, Oh 44106, USA. Ovulatory surges of human CG prevent hormone-induced granulosa cell tumor formation leading to the identification of tumor-associated changes in the transcriptome. *Mol Endocrinol* **16:** (6) 1230.

Poland J, Schadendorf D, Lage H, Schnolzer M, Celis JE, Sinha P*. 2002. *Univ Klinikum Charite, Inst Lab Med & Pathobiochem, Schumannstr 20-21, DE-10117 Berlin, Germany. Study of therapy resistance in cancer cells with functional proteome analysis. *Clin Chem Lab Med* **40:** (3) 221.

Robinson WH, Garren H, Utz PJ, Steinman L. 2002. Stanford Univ, Dept Neurol, Stanford, Ca 94305, USA. Proteomics for the development of DNA tolerizing vaccines to treat autoimmune disease. *Clin Immunol* **103:** (1) 7.

Rodi CP, Darnhofer-Patel B, Stanssens P, Zabeau M, Van den Boom D*. 2002. *SEQUENOM, 3595 John Hopkins Court, San Diego, Ca 92121, USA. A strategy for the rapid discovery of disease markers using the MassARRAY™ system. *Biotechniques* **32:** (Suppl) 62.

Saito-Hisaminato A, Katagiri T, Kakiuchi S, Nakamura T, Tsunoda T, Nakamura Y*. 2002. *Univ Tokyo, Ctr Human Genome, Minato ku, 4-6-1 Shirokanedai, Tokyo 108 8639, Japan. Genome-wide profiling of gene expression in 29 normal human tissues with a cDNA microarray. *DNA Res* **9:** (2) 35.

Schachter V. 2002. Hybrigenics, 3-5 Impasse Reille, FR-75014 Paris, France. Protein-interaction networks: From experiments to analysis. *Drug Discov Today* **7:** (11) S48.

Schwarz DA, Barry G, Mackay KB, Manu F, Naeve GS, Vana AM, Verge G, Conlon PJ, Foster AC, Maki RA. 2002. Neurocrine Biosci Inc, 10555 Sci Ctr Dr, San Diego, Ca 92121, USA. Identification of differentially expressed genes induced by transient ischemic stroke. *Mol Brain Res* **100:** (1-2) 12.

Sotiriou C, Khanna C, Jazaeri AA, Petersen D, Liu ET*. 2002. *Genome Inst Singapore, 1 Res Link, SG-117604 Singapore, Rep Singapore. Core biopsies can be used to distinguish differences in expression profiling by cDNA microarrays. *J Mol Diagn* **4:** (1) 30.

Suzuki H, Gabrielson E, Chen W, Anbazhagan R, Van Engeland M,

Copyright © 2002 John Wiley & Sons, Ltd. 

Weijenberg MP, Herman JG, Baylin SB*. 2002. *Johns Hopkins Univ, Kimmel Comprehens Canc Ctr, 1650 Orleans St, Baltimore, Md 21231, USA. A genomic screen for genes upregulated by demethylation and histone deacetylase inhibition in human colorectal cancer. *Nat Genet* **31:** (2) 141.

Suzuki J, Shen WJ, Nelson BD, Selwood SP, Murphy GM, Kanefara H, Takahashi S, Oida K, Miyamori I, Kraemer FB. 2002. Fukui Med Univ, III Dept Internal Med, Fukui 910 1193, Japan. Cardiac gene expression profile and lipid accumulation in response to starvation. *Am J Physiol* **283:** (1) E94.

Swindells MB, Overington JP. 2002. Inpharmatica, 60 Charlotte St, London W1T 2NU, England. Prioritizing the proteome: Identifying pharmaceutically relevant targets. *Drug Discov Today* **7:** (9) 516.

Thompson CJ, Tam NNC, Joyce JM, Leav I, Ho SM*. 2002. *Univ Massachusetts, Dept Surg, 55 Lake Ave Nth, Worcester, Ma 01655, USA. Gene expression profiling of testosterone and estradiol-17β-induced prostatic dysplasia in noble rats and response to the antiestrogen ICI 182,780. *Endocrinology* **143:** (6) 2093.

Todd R, Wong DTW. 2002. Harvard Sch Dent Med, Lab Oral & Maxillofacial Surg, 188 Longwood Ave, Boston, Ma 02115, USA. DNA hybridization arrays for gene expression analysis of human oral cancer. *J Dent Res* **81:** (2) 89.

Tyson KL, Weissberg PL, Shanahan CM*. 2002. *Addenbrookes Hosp, Dept Med, Addenbrookes Ctr Clin Invest, Hills Rd, Cambridge CB2 2QQ, England. Heterogeneity of gene expression in human atheroma unmasked using cDNA representational difference analysis. *Physiol Genomics* **9:** (2) 121.

Voit EO. 2002. Med Univ Sth Carolina, Dept Biometry & Epidemiol, 135 Cannon St, Charleston, SC 29425, USA. Metabolic modeling: A tool of drug discovery in the post-genomic era. *Drug Discov Today* **7:** (11) 621.

Yu LR, Johnson MD, Conrads TP, Smith RD, Morrison RS, Veenstra TD*. 2002. *NCI, SAIC Frederick, POB B, Frederick, Md 21702, USA. Proteome analysis of camptothecin-treated cortical neurons using isotope-coded affinity tags. *Electrophoresis* **23:** (11) 1591.

Zhang L, Zhang Y, Zhou YM, An S, Zhou YX, Cheng J*. 2002. *Tsing Hua Univ, Dept Biol Sci & Biotechnol, CN-100084 Beijing, Peoples Rep China. Response of gene expression in *Saccharomyces cerevisiae* to amphotericin B and nystatin measured by microarrays. *J Antimicrob Chemother* **49:** (6) 905.

### 8 EST, cDNA and other clone resources

Coimbra RS, Weil D, Brottier P, Blanchard S, Levi M, Hardelin JP, Weissenbach J, Petit C*. 2002. *Inst Pasteur, CNRS URA 1968, Unite Genet Deficits Sensoriels, FR-75724 Paris 15, France. A subtracted cDNA library from the zebrafish (*Danio rerio*) embryonic inner ear. *Genome Res* **12:** (6) 1007.

Fillingham JS, Chilcoat ND, Turkewitz AP, Orias E, Reith M, Pearlman RE*. 2002. *York Univ, Dept Biol, North York, Ontario, Canada M3J 1P3. Analysis of expressed sequence tags (ESTs) in the ciliated protozoan *Tetrahymena thermophila*. *J Eukaryot Microbiol* **49:** (2) 99.

Fung MC, Lau MT, Chen XG. 2002. Chinese Univ Hong Kong, Dept Biol, Shatin, Hong Kong, Peoples Rep China. Expressed sequence tag (EST) analysis of a *Schistosoma japonicum* cercariae cDNA library. *Acta Trop* **82:** (2) 215.

Kurth J, Varotto C, Pesaresi P, Biehl A, Richly E, Salamini F, Leister D*. 2002. *Max-Planck-Inst Zuchtungsforsch, Abt Pflanzenzuchtung & Ertragsphysiol, Carl von Linne Weg 10, DE-50829 Cologne, Germany. Gene-sequencing-tag expression analyses of 1,800 genes related to chloroplast functions. *Planta* **215:** (1) 101.

Martin SA, Caplice NC, Davey GC, Powell R*. 2002. *Natl Univ Ireland, Univ Coll Galway, Dept Microbiol, Galway, Rep Ireland. EST-based identification of genes expressed in the liver of adult Atlantic salmon (*Salmo salar*). *Biochem Biophys Res Commun* **293:** (1) 578.

Satou Y, Takatori N, Fujiwara S, Nishikata T, Saiga H, Kusakabe T, Shini T, Kohara Y, Satoh N*. 2002. *Kyoto Univ, Dept Zool, Sakyo ku, Kyoto 606 8502, Japan. *Ciona intestinalis* cDNA projects: Expressed sequence tag analyses and gene expression profiles during embryogenesis. *Gene* **287:** (1-2) 83.

### 9 Functional genomics

Baba T, Takeuchi F, Kuroda M, Yuzawa H, Aoki K, Oguchi A, Nagai Y, Iwama N, Asano K, Naimi T *et al*. 2002. c/o Hiramatsu K, Juntendo Univ, Dept Bacteriol, Bunkyo ku, 2-1-1 Hongo, Tokyo 113 8421, Japan. Genome and virulence determinants of high virulence community-acquired MRSA. *Lancet* **359:** (9320) 1819.

Benvenuti S, Cramer R, Bruce J, Waterfield MD, Jat PS*. 2002. *Royal Free & UCL Sch Med, Ludwig Inst Canc Res, 91 Riding House St, London W1W 7BS, England. Identification of novel candidates for replicative senescence by functional proteomics. *Oncogene* **21:** (28) 4403.

Bhattacharya D, Logue EC, Bakkour S, De Gregori J, Sha WC*. 2002. *Univ Calif Berkeley, Dept Mol & Cell Biol, 229 Stanley Hall, Berkeley, Ca 94720, USA. Identification of gene function by cyclical packaging rescue of retroviral cDNA libraries. *Proc Natl Acad Sci U S A* **99:** (13) 8838.

Dimopoulos G, Christophides GK, Meister S, Schultz J, White KP, Barillas-Mury C, Kafatos FC*. 2002. *European Mol Biol Lab, Meyerhofstr 1, DE-69117 Heidelberg, Germany. Genome expression analysis of *Anopheles gambiae*: Responses to injury, bacterial challenge, and malaria infection. *Proc Natl Acad Sci U S A* **99:** (13) 8814.

Heath LS, Ramakrishnan N*, Sederoff RR, Whetten RW, Chevone BI, Struble CA, Jouenne VY, Chen D, Van Zyl L, Grene R. 2002. *Virginia Tech, Dept Computer Sci, Blacksburg, Va 24061, USA. Studying the functional genomics of stress responses in loblolly pine with the Expresso microarray experiment management system. *Comp Funct Genom* **3:** (3) 226.

Kamoun S, Dong S, Hamada W, Huitema E, Kinney D, Morgan WR, Styer A, Testa A, Torto TA. 2002. Ohio State Univ, Dept Plant Pathol, Agr R&D Ctr, Wooster, Oh 44691, USA. From sequence to phenotype: Functional genomics of *Phytophthora*. *Can J Plant Pathol* **24:** (1) 6.

Kemmeren P, Van Berkum NL, Vilo J, Bijma T, Donders R, Brazma A, Holstege FCP*. 2002. *Univ Utrecht, Genomics Lab, POB 85060, NL-3508 AB Utrecht, The Netherlands. Protein interaction verification and functional annotation by integrated analysis of genome-scale data. *Mol Cell* **9:** (5) 1133.

Kendziorski CM, Cowley AW, Greene AS, Salgado HC, Jacob HJ, Tonellato PJ. 2002. Univ Wisconsin, Dept Biostat & Med Informat, 1300 Univ Ave, Madison, Wi 53706, USA. Mapping baroreceptor function to genome: A mathematical modeling approach. *Genetics* **160:** (4) 1687.

Kodama Y, Omura F, Takahashi K, Shirahige K, Ashikari T. 2002. Inst Fundamental Res, Suntory Res Ctr, 1-1-1 Wakayamadai, Shimamoto cho, Osaka 618 8503, Japan. Genome-wide expression analysis of genes affected by amino acid sensor Ssy1p in *Saccharomyces cerevisiae*. *Curr Genet* **41:** (2) 63.

Lin K, Kuang YY, Joseph JS, Kolatkar PR*. 2002. *Genome Inst Singapore, 1 Sci Pk Rd, SG-117528 Singapore, Rep Singapore. Conserved codon composition of ribosomal protein coding genes in *Escherichia coli*, *Mycobacterium tuberculosis* and *Saccharomyces cerevisiae*: Lessons from supervised machine learning in functional genomics. *Nucleic Acids Res* **30:** (11) 2599.

Robyr D, Suka Y, Xenarios I, Kurdistani SK, Wang A, Suka N, Grunstein M*. 2002. *UCLA, Dept Biol Chem, Boyer Hall, Los Angeles, Ca 90095, USA. Microarray deacetylation maps determine genome-wide functions for yeast histone deacetylases. *Cell* **109:** (4) 437.

Snel B, Bork P, Huynen MA. 2002. EMBL, Meyerhofstr 1, DE-69117 Heidelberg, Germany. The identification of functional modules from the genomic association of genes. *Proc Natl Acad Sci U S A* **99:** (9) 5890.

Vandepoele K, Raes J, DeVeylder L, Rouze P, Rombauts S, Inze D*. 2002. *State Univ Ghent, Dept Plant Syst Biol, Kl Ledeganckstr 35, BE-9000 Ghent, Belgium. Genome-wide analysis of core cell cycle genes in *Arabidopsis*. *Plant Cell* **14:** (4) 903.

Wang AG, Chen CH, Yang CW, Yen MY, Hsu WM, Liu JH, Fann MJ*. 2002. *Natl Yang Ming Univ, Inst Neurosci, Taipei 11221, Taiwan. Change of gene expression profiles in the retina following optic nerve injury. *Mol Brain Res* **100:** (1-2) 82.

Xie HQ, Wasserman A, Levine Z, Novik A, Grebinskiy V, Shoshan A,

Copyright © 2002 John Wiley & Sons, Ltd. 

Mintz L. 2002. Compugen Inc, 7 Ctr Dr, Jamiesburg, NJ 08831, USA. Large-scale protein annotation through gene ontology. *Genome Res* **12:** (5) 785.

Yamada M, Higuchi T. 2002. Showa Univ, Karasuyama Hosp, Dept Psychiat, Setagaya ku, 6-11-11 Kitakara Suyama, Tokyo 157 8577, Japan. Functional genomics and depression research beyond the monoamine hypothesis. *Eur Neuropsychopharmacol* **12:** (3) 235.

Zhen RG, Singh BK. 2001. BASF Plant Sci, POB 400, Princeton, NJ 08543, USA. From inhibitors to target site genes and beyond-herbicidal inhibitors as powerful tools for functional genomics. *Weed Sci* **49:** (2) 266.

### 10 Transcriptomics

Andrade PM, Silva IDCG, Borra RC, De Lima GR, Baracat EC. 2002. Univ Fed Sao Paulo, Avda Dr Altino Arantes 835, Apt 41, BR-04042-034 Sao Paulo, Brazil. Estrogen regulation of uterine genes *in vivo* detected by complementary DNA array. *Hormone Metab Res* **34:** (5) 238.

Bates MD, Erwin CR, Sanford LP, Wiginton D, Bezerra JA, Schatzman LC, Jegga AG, Ley-Ebert C, Williams SS, Steinbrecher KA *et al.* 2002. Childrens Hosp, Div Pediat Gastroenterol Hepatol & Nutr, 3333 Burnet Ave, Cincinnati, Oh 45229, USA. Novel genes and functional relationships in the adult mouse gastrointestinal tract, identified by microarray analysis. *Gastroenterology* **122:** (5) 1467.

Bray EA. 2002. Univ Calif Riverside, Dept Bot & Plant Sci, Riverside, Ca 92521, USA. Classification of genes differentially expressed during water-deficit stress in *Arabidopsis thaliana*: An analysis using microarray and differential expression data. *Ann Bot* **89:** 803.

Cheval L, Virlon B, Billon E, Aude JC, Elalouf JM, Doucet A*. 2002. *CE Saclay, Batiment 520, FR-91198 Gif-sur-Yvette, France. Large-scale analysis of gene expression: Methods and application to the kidney. *J Nephrol* **15:** (Suppl 5) S170.

Chin KV, Seifer DB, Feng B, Lin Y, Shih WC. 2002. Univ Med & Dent New Jersey, Canc Inst, 195 Little Albany St, New Brunswick, NJ 08901, USA. DNA microarray analysis of the expression profiles of luteinized granulosa cells as a function of ovarian reserve. *Fertil Steril* **77:** (6) 1214.

Clark TA, Sugnet CW, Ares M*. 2002. *Univ Calif, Dept Mol Cell & Dev Biol, Santa Cruz, Ca 95064, USA. Genomewide analysis of mRNA processing in yeast using splicing-specific microarrays. *Science* **296:** (5569) 907.

Colebatch G, Kloska S, Trevaskis B, Freund S, Altmann T, Udvardi MK*. 2002. *Max Planck Inst Mol Plant Physiol, Muhlenberg 1, DE-14476 Golm, Germany. Novel aspects of symbiotic nitrogen fixation uncovered by transcript profiling with cDNA arrays. *Mol Plant Microbe Interact* **15:** (5) 411.

Draghici S. 2002. Wayne State Univ, Dept Comp Sci, 431 State Hall, Detroit, Mi 48202, USA. Statistical intelligence: Effective analysis of high-density microarray data. *Drug Discov Today* **7:** (11) S55.

Fischer MD, Gorospe JR, Felder E, Bogdanovich S, Pedrosa-Domellof F, Ahima RS, Rubinstein NA, Hoffman EP, Khurana TS*. 2002. *Univ Penn, Dept Physiol, 3700 Hamilton Walk, Philadelphia, Pa 19104, USA. Expression profiling reveals metabolic and structural components of extraocular muscles. *Physiol Genomics* **9:** (2) 71.

Gerhold DL, Liu F, Jiang G, Li Z, Xu J, Lu M, Sachs JR, Bagchi A, Fridman A, Holder DJ *et al.* 2002. c/o Zhang BB, Merck Res Labs, Dept Mol Endocrinol, 126 East Lincoln Ave, Rahway, NJ 07065, USA. Gene expression profile of adipocyte differentiation and its regulation by peroxisome proliferator-activated receptor-γ agonists. *Endocrinology* **143:** (6) 2106.

Gill RT, Katsoulakis E, Schmitt W, Taroncher-Oldenburg G, Misra J, Stephanopoulos G*. 2002. *MIT, Dept Chem Engn, 77 Massachusetts Ave, Cambridge, Ma 02139, USA. Genome-wide dynamic transcriptional profiling of the light-to-dark transition in *Synechocystis* sp strain PCC 6803. *J Bacteriol* **184:** (13) 3671.

Gill RT, Wildt S, Yang YT, Ziesman S, Stephanopoulos G*. 2002. *Address as above. Genome-wide screening for trait conferring genes using DNA microarrays. *Proc Natl Acad Sci U S A* **99:** (10) 7033.

Han X, Bolcato AL, Amar S*. 2002. *Boston Univ, Dept Periodontol & Oral Biol, 100 East Newton St, Boston, Ma 02215, USA. Identification of genes differentially expressed in cultured human osteoblasts *versus* human fibroblasts by DNA microarray analysis. *Connect Tissue Res* **43:** (1) 63.

Hanano S, Amagai M, Kaneko T, Kuwata C, Tsugane T, Sakurai N, Nakamura Y, Shibata D*, Tabata S. 2002. *Kazusa DNA Res Inst, 1532-3 Yana, Chiba 292 0812, Japan. Analysis of gene expression in *Arabidopsis thaliana* by array hybridization with genomic DNA fragments aligned along chromosomal regions. *Plant J* **30:** (2) 247.

Hobson RJ, McBride AJA, Kempsell KE, Dale JW*. 2002. *Univ Surrey, Sch Biomed & Life Sci, Guildford GU2 7XH, England. Use of an arrayed promoter-probe library for the identification of macrophage-regulated genes in *Mycobacterium tuberculosis*. *Microbiology* **148:** (5) 1571.

Kao LC, Tulac S, Lobo S, Imani B, Yang JP, Germeyer A, Osteen K, Taylor RN, Lessey BA, Giudice LC*. 2002. *Stanford Univ, Div Reprod Endocrinol & Infertil, Stanford, Ca 94305, USA. Global gene profiling in human endometrium during the window of implantation. *Endocrinology* **143:** (6) 2119.

Kroll TC, Wolfl S*. 2002. *Klinikum Friedrich Schiller Univ, Innere Med Klin, AG Mol Biol, Erlanger Allee 101, DE-07747 Jena, Germany. Ranking: A closer look on globalisation methods for normalisation of gene expression arrays (Online paper). *Nucleic Acids Res* **30:** (11) E50.

Menssen A, Hermeking H*. 2002. *Max-Planck-Inst Biochem, Independent Junior Res Grp, Klopferspitz 18A, DE-82152 Martinsried, Germany. Characterization of the c-MYC-regulated transcriptome by SAGE: Identification and analysis of c-MYC target genes. *Proc Natl Acad Sci U S A* **99:** (9) 6274.

Nakamoto T, Suzuki T, Huang JH, Matsumura T, Seo S, Honda H, Sakai R, Hirai H*. 2002. *Univ Tokyo, Dept Hematol & Oncol, Bunkyo ku, 7-3-1 Hongo, Tokyo 113 8655, Japan. Analysis of gene expression profile in $p130^{Cas}$-deficient fibroblasts. *Biochem Biophys Res Commun* **294:** (3) 635.

Narasimhan S, Santiago F, Koski RA, Anderson JF, Brei B, Fish D, Fikrig E*. 2002. *Yale Univ, Dept Internal Med, 333 Cedar St, New Haven Ct 06520, USA. Examination of the *Borrelia burgdorferi* transcriptome in *Ixodes scapularis* during feeding. *J Bacteriol* **184:** (11) 3122.

Paustian ML, May BJ, Kapur V*. 2002. *Univ Minnesota, Biomed Genom Ctr, 1971 Commonwealth Ave, St Paul, Mn 55108, USA. Transcriptional response of *Pasteurella multocida* to nutrient limitation. *J Bacteriol* **184:** (13) 3734.

Pletcher SD, Macdonald SJ, Marguerie R, Certa U, Stearns SC, Goldstein DB, Partridge L. 2002. UCL, Dept Biol, Galton Lab, Darwin Bldg, Gower St, London WC1E 6BT, England. Genome-wide transcript profiles in aging and calorically restricted *Drosophila melanogaster*. *Curr Biol* **12:** (9) 712.

Saha S, Sparks AB, Rago C, Akmaev V, Wang CJ, Vogelstein B, Kinzler KW*, Velculescu VE. 2002. *Howard Hughes Med Inst, Baltimore, Md 21231, USA. Using the transcriptome to annotate the genome. *Nat Biotechnol* **20:** (5) 508.

Shaulsky G, Loomis WF*. 2002. *Univ Calif San Diego, Div Biol, La Jolla, Ca 92093, USA. Gene expression patterns in *Dictyostelium* using microarrays. *Protist* **153:** (2) 93.

Sironen RK, Karjalainen HM, Torronen K, Elo MA, Kaarniranta K, Takigawa M, Helminen HJ, Lammi MJ*. 2002. *Univ Kuopio, Dept Anat, POB 1627, FI-70211 Kuopio, Finland. High pressure effects on cellular expression profile and mRNA stability. A cDNA array analysis. *Biorheology* **39:** (1-2) 111.

Sugiyama T, Ishii S, Yamamoto J, Irie R, Saito K, Otuki T, Wakamatsu A, Suzuki Y, Hio Y, Ota T *et al.* 2002. Kyowa Hakko Kogyo Co Ltd, 3-6-6 Asahi cho, Machida, Tokyo 194 8533, Japan. cDNA macroarray analysis of gene expression in synoviocytes stimulated with TNFα. *FEBS Lett* **517:** (1-3) 121.

Swidzinski JA, Sweetlove LJ, Leaver CJ*. 2002. *Univ Oxford, Dept Plant Sci, Sth Parks Rd, Oxford OX1 3RB, England. A custom microarray analysis of gene expression during programmed cell death in *Arabidopsis thaliana*. *Plant J* **30:** (4) 431.

Tadjali M, Seidelin JB, Olsen J, Troelsen JT*. 2002. *Univ Copenhagen, Dept Med Biochem & Genet, Blegdamsvej 3, DK-2200 Copenhagen N, Denmark. Transcriptome changes during intestinal cell differentiation. *Biochim Biophys Acta* **1589:** (2) 160.

Copyright © 2002 John Wiley & Sons, Ltd. 

Trotter SA, Brill LB, Bennett JP*. 2002. *Univ Virginia, POB 800394, Charlottesville, Va 22908, USA. Stability of gene expression in post-mortem brain revealed by cDNA gene array analysis. *Brain Res* **942:** (1-2) 120.

Van Ruissen F, Jansen BJH, De Jongh GJ, Zeeuwen PLJM, Schalkwijk J. 2002. Acad Med Ctr, Neurozintuigen Lab, POB 22660, NL-1100 DD Amsterdam, The Netherlands. A partial transcriptome of human epidermis. *Genomics* **79:** (5) 671.

Watanabe H, Suzuki A, Mizutani T, Khono S, Lubahn DB, Handa H, Iguchi T*. 2002. *Okazaki Natl Res Inst, Ctr Integrat Biosci & Core Res Evolut Sci & Tech, 38 Nishigonaka, Aichi 444 8585, Japan. Genome-wide analysis of changes in early gene expression induced by oestrogen. *Genes Cells* **7:** (5) 497.

Yonehara K, Suzuki M, Nishihara M*. 2002. *Univ Tokyo, Dept Vet Physiol, Bunkyo ku, 1-1-1 Yayoi, Tokyo 113 8657, Japan. Sex-related differences in gene expression in neonatal rat hypothalamus assessed by cDNA microarray analysis. *Endocr J* **49:** (2) 131.

### 11 Proteomics

Amiour N, Merlino M, Leroy P, Branlard G*. 2002. *UMR-ASP-UBP, 234 Ave Brezet, FR-63039 Clermont Ferrand 02, France. Proteomic analysis of amphiphilic proteins of hexaploid wheat kernels. *Proteomics* **2:** (6) 632.

Antelmann H, Yamamoto H, Sekiguchi J, Hecker M. 2002. Univ Greifswald, Inst Mikrobiol, FL Jahnstr 15, DE-17487 Greifswald, Germany. Stabilization of cell wall proteins in *Bacillus subtilis*: A proteomic approach. *Proteomics* **2:** (5) 591.

Barcelo-Batllori S, Andre M, Servis C, Levy N, Takikawa O, Michetti P, Reymond M, Felley-Bosco E*. 2002. *Inst Pharmacol & Toxicol, rue Bugnon 27, CH-1005 Lausanne, Switzerland. Proteomic analysis of cytokine induced proteins in human intestinal epithelial cells: Implications for inflammatory bowel diseases. *Proteomics* **2:** (5) 551.

Beranova-Giorgianni S, Giorgianni F, Desiderio DM*. 2002. *Univ Tennessee, CB Stout Neurosci MS Lab, Ctr Hlth Sci, 847 Monroe Ave, Memphis, Tn 38163, USA. Analysis of the proteome in the human pituitary. *Proteomics* **2:** (5) 534.

Bernardo K, Fleer S, Pakulat N, Krut O, Hunger F, Kronke M. 2002. Univ Cologne, Med Ctr, Inst Med Microbiol Immunol & Hyg, Goldenfelsstr 19-21, DE-50935 Cologne, Germany. Identification of *Staphylococcus aureus* exotoxins by combined sodium dodecyl sulfate gel electrophoresis and matrix-assisted laser desorption/ionization-time of flight mass spectrometry. *Proteomics* **2:** (6) 740.

Bernardo K, Pakulat N, Macht M, Krut O, Seifert H, Fleer S, Hunger F, Kronke M. 2002. Address as above. Identification and discrimination of *Staphylococcus aureus* strains using matrix-assisted laser desorption/ionization-time of flight mass spectrometry. *Proteomics* **2:** (6) 747.

Boxman ILA, Hensbergen PJ, Van der Schors RC, Bruynzeel DP, Tensen CP, Ponec M*. 2002. *Sylvius Labs, Dept Dermatol, Wassenaarseweg 72, NL-2333 AL Leiden, Netherlands. Proteomic analysis of skin irritation reveals the induction of HSP27 by sodium lauryl sulphate in human skin. *Br J Dermatol* **146:** (5) 777.

Bumann D, Aksu S, Wendland M, Janek K, Zimny-Arndt U, Sabarth N, Meyer TF*, Jungblut PR. 2002. *Max-Planck-Inst Infekt Biol, Mol Biol Abt, Schumannstr 21-22, DE-10117 Berlin, Germany. Proteome analysis of secreted proteins of the gastric pathogen *Helicobacter pylori*. *Infect Immun* **70:** (7) 3396.

Carrascal M, Carujo S, Bachs O, Abian J*. 2002. *IIBB- CSIC-IDIBAPS, Dept Med Bioanal Struct & Biol Mass Spectrometry, Rosello 161 7a, ES-08036 Barcelona, Spain. Identification of $p21^{Cip1}$ binding proteins by gel electrophoresis and capillary liquid chromatography microelectrospray tandem mass spectrometry. *Proteomics* **2:** (4) 455.

Chivasa S, Ndimba BK, Simon WJ, Robertson D, Yu XL, Yu XL, Knox JP, Bolwell P, Slabas AR*. 2002. *Univ Durham, Dept Biol Sci, Integrat Cell Biol Lab, Durham DH1 3LE, England. Proteomic analysis of the *Arabidopsis thaliana* cell wall. *Electrophoresis* **23:** (11) 1754.

Cho MJ, Jeon BS, Park JW, Jung TS, Song JY, Lee WK, Choi YJ, Choi SH, Park SG, Park JU *et al.* 2002. c/o Rhee KH, Gyeongsang Natl Univ, Dept Microbiol, 90 Chilam dong, Chinju 660750, Kyung nam, South Korea. Identifying the major proteome components of *Helicobacter pylori* strain 26695. *Electrophoresis* **23:** (7-8) 1161.

Conrads TP, Issaq HJ, Veenstra TD*. 2002. *NCI, SAIC Frederick Inc, POB 8, Frederick, Md 21701, USA. An accurate mass tag strategy for quantitative and high throughput proteome measurements. *Instrum Sci Technol* **30:** (2) 211.

Drews O, Weiss W, Reil G, Parlar H, Wait R, Gorg A*. 2002. *Tech Univ Munich, FG Proteomik, Forum 2, DE-85350 Freising Weihenstephan, Germany. High pressure effects step-wise altered protein expression in *Lactobacillus sanfranciscensis*. *Proteomics* **2:** (6) 765.

Gohar M, Okstad OA, Gilois N, Sanchis V, Kolsto AB, Lereclus D. 2002. INRA, Unite Rech Lutte Biol, FR-78285 Guyancourt, France. Two-dimensional electrophoresis analysis of the extracellular proteome of *Bacillus cereus* reveals the importance of the PlcR regulon. *Proteomics* **2:** (6) 784.

Griese M, Noss J, Von Bredow C. 2002. Univ Munich, Childrens Hosp, Pettenkoferstr 8a, DE-80336 Munich, Germany. Protein pattern of exhaled breath condensate and saliva. *Proteomics* **2:** (6) 690.

Hommais F, Laurent-Winter C, Labas V, Krin E, Tendeng C, Soutourina O, Danchin A, Bertin P*. 2002. *SCK/CEN, Radioact Waste & Clean Up Div, Lab Microbiol, Boeretang 200, BE-2400 Mol, Belgium. Effect of mild acid pH on the functioning of bacterial membranes in *Vibrio cholerae*. *Proteomics* **2:** (5) 571.

Isfort RJ, Wang F, Greis KD, Sun YP, Keough TW, Farrar RP, Bodine SC, Anderson NL. 2002. Procter & Gamble Pharmaceut, Hlth Care Res Ctr, Div Res, 8700 Mason Montgomery Rd, Mason, Oh 45040, USA. Proteomic analysis of rat soleus muscle undergoing hindlimb suspension-induced atrophy and reweighting hypertrophy. *Proteomics* **2:** (5) 543.

Jaubert S, Ledger TN, Laffaire JB, Piotte C, Abad P, Rosso MN*. 2002. *INRA, Unite Interact Plantes Microorganismes & Sante Vegetale, 123 Blvd Francis Meilland, FR-06606 Antibes, France. Direct identification of stylet secreted proteins from root-knot nematodes by a proteomic approach. *Mol Biochem Parasitol* **121:** (2) 205.

Jimenez CR, Eyman M, Lavina ZS, Gioio A, Li KW, Van der Schors RC, Geraerts WPM, Giuditta A, Kaplan BB, Van Minnen J*. 2002. *Vrije Univ Amsterdam, Neurosci Res Inst, Amsterdam, The Netherlands. Protein synthesis in synaptosomes: A proteomics analysis. *J Neurochem* **81:** (4) 735.

Kettman JR, Coleclough C, Frey JR, Lefkovits I*. 2002. *Univ Basel, Inst Physiol, Vesalgasse 1, CH-4051 Basel, Switzerland. Clonal proteomics: One gene - family of proteins. *Proteomics* **2:** (6) 624.

Kim HJ, Song EJ, Lee KJ*. 2002. *EWHA Womans Univ, Div Mol Life Sci, Seoul 120750, South Korea. Proteomic analysis of protein phosphorylations in heat shock response and thermotolerance. *J Biol Chem* **277:** (26) 23193.

Knepper MA. 2002. NIH/NHLBI, Kidney & Electrolyte Metab Lab, 10 Ctr Dr, MSC 1603, Bldg 10, Rm 6N260, Bethesda Md 20892, USA. Proteomics and the kidney. *J Am Soc Nephrol* **13:** (5) 1398.

Kornilovska I, Nilsson I*, Utt M, Ljungh A, Wadstrom T. 2002. *Lund Univ, Dept Med Microbiol Dermatol & Infect, Solvegatan 23, SE-22362 Lund, Sweden. Immunogenic proteins of *Helicobacter pullorum, Helicobacter bilis* and *Helicobacter hepaticus* identified by two-dimensional gel electrophoresis and immunoblotting. *Proteomics* **2:** (6) 775.

Lichtenfels R, Kellner R, Bukur J, Beck J, Brenner W, Ackermann A, Seliger B*. 2002. *Univ Mainz, Div Internal Med 3, Langenbeckstr 1, DE-55101 Mainz, Germany. Heat shock protein expression and anti-heart shock protein reactivity in renal cell carcinoma. *Proteomics* **2:** (5) 561.

Lock RA, Coombs GW, McWilliams TM, Pearman JW, Grubb WB, Melrose GJH, Forbes GM. 2002. Royal Perth Hosp, Dept Microbiol & Infect Dis, Perth, WA 6000, Australia. Proteome analysis of highly immunoreactive proteins of *Helicobacter pylori*. *Helicobacter* **7:** (3) 175.

Maguire PB, Wynne KJ, Harney DF, O'Donoghue NM, Stephens G, Fitzgerald DJ*. 2002. *Royal Coll Surgeons Ireland, Dept Clin Pharmacol, 123 St Stephens Green, Dublin 2, Rep Ireland. Identification of the phosphotyrosine proteome from thrombin activated platelets. *Proteomics* **2:** (6) 642.

Malmstrom J, Larsen K, Hansson L, Lofdahl CG, Norregard-Jensen O,

Copyright © 2002 John Wiley & Sons, Ltd. 

Marko-Verga G*, Westergren-Thorsson G. 2002. *Lund Univ, SE-22100 Lund, Sweden. Proteoglycan and proteome profiling of central human pulmonary fibrotic tissue utilizing miniaturized sample preparation: A feasibility study. *Proteomics* **2:** (4) 394.

Marceau A, Zagorec M, Champomier-Verges M*. 2002. *INRA, Ctr Rech Jouy-en-Josas, Unite Flore Lactique & Environm Carne, FR-78350 Jouy-en-Josas, France. Analysis of *Lactobacillus sakei* adaptation to its environment by a proteomic approach. *Sci Aliment* **22:** (1-2) 97.

Molestina RE, Klein JB, Miller RD, Pierce WH, Ramirez JA, Summersgill JT*. 2002. *Univ Louisville, Div Infect Dis, Instructional Bldg, Louisville, Ky 40292, USA. Proteomic analysis of differentially expressed *Chlamydia pneumoniae* genes during persistent infection of HEp-2 cells. *Infect Immun* **70:** (6) 2976.

Mullaney BP, Marks JD, Pallavicini MG*. 2002. *UCSF Cancer Ctr, 2340 Sutter St, Box 0808, San Francisco, Ca 94115, USA. Mimotopes and proteome analyses using human genomic and cDNA epitope phage display. *Comp Funct Genom* **3:** (3) 254.

Neumann M, Von Bredow C, Ratjen F, Griese M. 2002. Univ Munich, Children's Hosp, Pettenkoferstr 8A, DE-80336 Munich, Germany. Bronchoalveolar lavage protein patterns in children with malignancies, immunosuppression, fever and pulmonary infiltrates. *Proteomics* **2:** (6) 683.

Oguri T, Takahata I, Katsuta K, Nomura E, Hidaka M, Inagaki N*. 2002. *Nara Inst Sci & Technol, Div Signal Transduct, Ikoma 630 0101, Japan. Proteome analysis of rat hippocampal neurons by multiple large gel two-dimensional electrophoresis. *Proteomics* **2:** (6) 666.

Oosthuizen MC, Steyn B, Theron J, Cosette P, Lindsay D, Von Holy A, Brozel VS. 2002. Univ Pretoria, Lab Biofilm Physiol, ZA-0002 Pretoria, Rep Sth Africa. Proteomic analysis reveals differential protein expression by *Bacillus cereus* during biofilm formation. *Appl Environ Microbiol* **68:** (6) 2770.

Ostergaard O, Melchior S, Roepstorff P, Svensson B*. 2002. *Carlsberg Lab, Dept Chem, Gamle Carlsberg Vej 10, DK-2500 Copenhagen, Denmark. Initial proteome analysis of mature barley seeds and malt. *Proteomics* **2:** (6) 733.

Rabilloud T, Heller M, Gasnier F, Luche S, Rey C, Aebersold R, Benahmed M, Louisot P, Lunardi J. 2002. CEA-Grenoble, DRDC/BECP, EA UJF 294, Lab Bioenerget Cellulaire & Pathol, 17 rue Martyrs, FR-38054 Grenoble 9, France. Proteomics analysis of cellular response to oxidative stress: Evidence for *in vivo* overoxidation of peroxiredoxins at their active site. *J Biol Chem* **277:** (22) 19396.

Rabus R, Gade D, Helbig R, Bauer M, Glockner FO, Kube M, Schlesner H, Reinhardt R, Amann R. 2002. Max-Planck-Inst Marine Mikrobiol, Celsiusstr 1, DE-28359 Bremen, Germany. Analysis of *N*-acetylglucosamine metabolism in the marine bacterium *Pirellula* sp strain 1 by a proteomic approach. *Proteomics* **2:** (6) 649.

Rosato V, Pucello N, Giuliano G. 2002. ENEA, Biotechnol Unit, Casaccia Res Ctr, IT-00100 Rome, Italy. Evidence for cysteine clustering in thermophilic proteomes. *Trends Genet* **18:** (6) 278.

Salekdeh GH, Siopongco J, Wade LJ, Ghareyazie B, Bennett J*. 2002. *Int Rice Res Inst, DAPO Box 7777, Manila, Philippines. A proteomic approach to analyzing drought- and salt-responsiveness in rice. *Field Crop Res* **76:** (2-3) 199.

Sauter ER, Zhu W, Fan XJ, Wassell RP, Chervoneva I, Du Bois GC. 2002. Thomas Jefferson Univ, Dept Surg, 1025 Walnut St, Philadelphia, Pa 19107, USA. Proteomic analysis of nipple aspirate fluid to detect biologic markers of breast cancer. *Br J Cancer* **86:** (9) 1440.

Schlosser A, Lehmann WD*. 2002. *German Canc Res Ctr, Cent Spect Unit, Neuenheimer Field 280, DE-69120 Heidelberg, Germany. Patchwork peptide sequencing: Extraction of sequence information from accurate mass data of peptide tandem mass spectra recorded at high resolution. *Proteomics* **2:** (5) 524.

Shimizu T, Shima K, Yoshino K, Yonezawa K, Shimizu T, Hayashi H*. 2002. *Univ Tsukuba, Dept Microbiol, 1-1-1 Ten-nohdai, Tsukuba, Ibaraki 305 8575, Japan. Proteome and transcriptome analysis of the virulence genes regulated by the VirR/VirS system in *Clostridium perfringens*. *J Bacteriol* **184:** (10) 2587.

Skold K, Svensson M, Kaplan A, Bjorkesten L, Astrom J, Andren PE*. 2002. *Univ Uppsala, Dept Pharmaceut Biosci, Med & Biol Mass Spectrometry Ctr, SE-75124 Uppsala, Sweden. A neuroproteomic approach to targeting neuropeptides in the brain. *Proteomics* **2:** (4) 447.

Tilleman K, Van den Haute C, Geerts H, Van Leuven F, Esmans EL, Moens L*. 2002. *Univ Instelling Antwerp, Dept Biochem, Universiteitspl 1, BE-2610 Antwerp, Belgium. Proteomics analysis of the neurodegeneration in the brain of $\tau$ transgenic mice. *Proteomics* **2:** (6) 656.

Tremoulet F, Duche O, Namane A, Martinie B, Labadie JC*. 2002. *INRA, Lab Microbiol, Rech Viande Stn, FR-63122 St Genes Champanelle, France. Comparison of protein patterns of *Listeria monocytogenes* grown in biofilm or in planktonic mode by proteomic analysis. *FEMS Microbiol Lett* **210:** (1) 25.

Ueberle B, Frank R, Herrmann R*. 2002. *Univ Heidelberg, Zentrum Mol Biol, Neuenheimer Feld 282, DE-69120 Heidelberg, Germany. The proteome of the bacterium *Mycoplasma pneumoniae*: Comparing predicted open reading frames to identified gene products. *Proteomics* **2:** (6) 754.

Vytvytska O, Nagy E, Bluggel M, Meyer HE, Kurzbauer R, Huber LA, Klade CS*. 2002. *InterCell AG, Rennweg 95B, AU-1030 Vienna, Austria. Identification of vaccine candidate antigens of *Staphylococcus aureus* by serological proteome analysis. *Proteomics* **2:** (5) 580.

Yuan XL, Russell T, Wood G, Desiderio DM*. 2002. *Univ Tennessee, Charles B Stout Neurosci Mass Spect Lab, Ctr Hlth Sci, Memphis, Tn 38163, USA. Analysis of the human lumbar cerebrospinal fluid proteome. *Electrophoresis* **23:** (7-8) 1185.

### 12 Protein structural genomics

Chakravarty S, Varadarajan R*. 2002. *Indian Inst Sci, Mol Biophys Unit, IN-560012 Bangalore, India. Elucidation of factors responsible for enhanced thermal stability of proteins: A structural genomics based study. *Biochemistry* **41:** (25) 8152.

Chatterjee A, Bhavesh NS, Panchal SC, Hosur RV*. 2002. *Tata Inst Fundamental Res, Dept Chem Sci, Homi Bhabha Rd, IN-400005 Bombay, India. A novel protocol based on HN(C)N for rapid resonance assignment in ($^{15}$N,$^{13}$C) labeled proteins: Implications to structural genomics. *Biochem Biophys Res Commun* **293:** (1) 427.

Kihara D, Zhang Y, Lu H, Kolinski A, Skolnick J*. 2002. *Danforth Plant Sci Ctr, Lab Computat Genomics, 975 Nth Warson Rd, St Louis, Mo 63132, USA. *Ab initio* protein structure prediction to a genomic scale: Application to the *Mycoplasma genitalium* genome. *Proc Natl Acad Sci U S A* **99:** (9) 5993.

### 13 Metabolomics

Papin JA, Price ND, Edwards JS, Palsson BO*. 2002. *Univ Calif San Diego, Dept Bioengn, 9500 Gilman Dr, La Jolla, Ca 92093, USA. The genome-scale metabolic extreme pathway structure in *Haemophilus influenzae* shows significant network redundancy. *J Theor Biol* **215:** (1) 67.

Price ND, Papin JA, Palsson BO*. 2002. *Univ Calif San Diego, Dept Bioengn, La Jolla, Ca 92093, USA. Determination of redundancy and systems properties of the metabolic network of *Helicobacter pylori* using genome-scale extreme pathway analysis. *Genome Res* **12:** (5) 760.

Rosenkrands I, Slayden RA, Crawford J, Aagaard C, Barry BE, Andersen P. 2002. Statens Serum Inst, Dept TB Immunol, 5 Artillerivej, DK-2300 Copenhagen S, Denmark. Hypoxic response of *Mycobacterium tuberculosis* studied by metabolic labeling and proteome analysis of cellular and extracellular proteins. *J Bacteriol* **184:** (13) 3485.

Yang C, Hua Q, Shimizu K*. 2002. *Kyushu Inst Technol, Dept Biochem Engn & Sci, Iizuka, Fukuoka 820 8502, Japan. Integration of the information from gene expression and metabolic fluxes for the analysis of the regulatory mechanisms in *Synechocystis*. *Appl Microbiol Biotechnol* **58:** (6) 813.

### 14 Genomic approaches to development

Chen HW, Chen JJW, Tzeng CR*, Li HN, Chang SJ, Cheng YF, Chang CW, Wang RS, Yang PC, Lee YT. 2002. *Taipei Med Univ Hosp, Dept Obstet & Gynecol, 252 Wu Hsing St, Taipei, Taiwan. Global

Copyright © 2002 John Wiley & Sons, Ltd. 

analysis of differentially expressed genes in early gestational decidua and chorionic villi using a 9600 human cDNA microarray. *Mol Hum Reprod* **8:** (5) 475.

Costouros NG, Libutti SK*. 2002. *NIH, Bldg 10, Room 3C428, Bethesda, Md 20892, USA. Microarray technology and gene expression analysis for the study of angiogenesis. *Exp Opin Biol Ther* **2:** (5) 545.

Golling G, Amsterdam A, Sun ZX, Antonelli M, Maldonado E, Chen WB, Burgess S, Haldi M, Artzt K, Farrington S *et al*. 2002. c/o Hopkins N, MIT, Ctr Canc Res, 77 Massachusetts Ave, Cambridge, Ma 02139, USA. Insertional mutagenesis in zebrafish rapidly identifies genes essential for early vertebrate development. *Nat Genet* **31:** (2) 135.

Harafuji N, Keys DN, Levine M*. 2002. *Univ Calif, Div Genet & Dev, 229 Stanley Hall, Berkeley, Ca 94720, USA. Genome-wide identification of tissue-specific enhancers in the *Ciona* tadpole. *Proc Natl Acad Sci U S A* **99:** (10) 6802.

Kato HD, Terao Y, Ogawa M, Matsuda T, Arima T, Kato K, Yong Z, Wake N. 2002. Kyushu Univ, Dept Mol Genet, Tsurumihara 4546, Beppu, Oita 874 0838, Japan. Growth-associated gene expression profiles by microarray analysis of trophoblast of molar pregnancies and normal villi. *Int J Gynecol Pathol* **21:** (3) 255.

Master SR, Hartman JL, D'Cruz CM, Moody SE, Keiper EA, Ha SI, Cox JD, Belka GK, Chodosh LA*. 2002. *Univ Penn, Dept Canc Biol, 421 Curie Blvd, Philadelphia, Pa 19104, USA. Functional microarray analysis of mammary organogenesis reveals a developmental role in adaptive thermogenesis. *Mol Endocrinol* **16:** (6) 1185.

Sha JH, Zhou ZM, Li JM, Yin LL, Yang HM, Hu GX, Luo M, Chan HC, Zhou KY. 2002. Nanjing Med Univ, Lab Reprod Med, CN-210029 Nanjing, Peoples Rep China. Identification of testis development and spermatogenesis-related genes in human and mouse testes using cDNA arrays. *Mol Hum Reprod* **8:** (6) 511.

Zinselmeier C, Sun YJ, Helentjaris T, Beatty M, Yang S, Smith H, Habben J. 2002. Pioneer Hibred Int Inc, Dept Res & Prod Dev, 7250 NW 62nd Ave, Johnston, Ia 50131, USA. The use of gene expression profiling to dissect the stress sensitivity of reproductive development in maize. *Field Crop Res* **75:** (2-3) 111.

Zou JW, Sun MX*, Yang HY. 2002. *Wuhan Univ, Key Lab Minist Educ Plant Dev Biol, CN-430072 Wuhan, Peoples Rep China. Single-embryo RT-PCR assay to study gene expression dynamics during embryogenesis in *Arabidopsis thaliana*. *Plant Mol Biol Rep* **20:** (1) 19.

### 15 Technological advances

Anbazhagan R. 2002. Dept Pathol, 418 Nth Bond St, Suite 301, Baltimore, Md 21231, USA. Microarray Assistant clone organizer and array simulator. *Biotechniques* **32:** (6) 1398.

Andersson T, Unneberg P, Nilsson P, Odeberg J, Quackenbush J, Lundeberg J*. 2002. *Royal Inst Technol, SCFAB, Dept Biotechnol, SE-10691 Stockholm, Sweden. Monitoring of representational difference analysis subtraction procedures by global microarrays. *Biotechniques* **32:** (6) 1348.

Berggren KN, Schulenberg B, Lopez MF, Steinberg TH, Bogdanova A, Smejkal G, Wang A, Patton WF*. 2002. *Mol Probes Inc, Proteomics Sect, 4849 Pitchford Ave, Eugene, Or 97402, USA. An improved formulation of SYPRO Ruby protein gel stain: Comparison with the original formulation and with a ruthenium II tris (bathophenanthroline disulfonate) formulation. *Proteomics* **2:** (5) 486.

Bergkvist J, Ekstrom S, Wallman L, Lofgren M, Marko-Varga G, Nilsson J, Laurell T*. 2002. *Lund Univ, Dept Elect Measurements, POB 118, SE-22100 Lund, Sweden. Improved chip design for integrated solid-phase microextraction in on-line proteomic sample preparation. *Proteomics* **2:** (4) 422.

Chan PJ, Mann SL, Corselli JU, Patton WC, King A, Jacobson JD. 2002. Loma Linda Univ, Ctr Fertil, 11370 Anderson St, Loma Linda, Ca 92354, USA. A simple DNA disc chip in a microarray design based on modified comparative genomic hybridization for analysis. *Fertil Steril* **77:** (5) 1056.

Chapman K. 2002. Ciphergen Biosyst Ltd, Surrey Technol Ctr, Surrey Res Pk, Guildford GU2 7YG, England. The ProteinChip® Biomarker System from Ciphergen Biosystems: A novel proteomics platform for rapid biomarker discovery and validation. *Biochem Soc Trans* **30:** (Apr) 82.

Chayen NE, Saridakis E. 2002. Univ London, Imperial Coll Sci Technol & Med, Div Biomed Sci, London SW7 2AZ, England. Protein crystallization for genomics: Towards high-throughput optimization techniques. *Acta Crystallogr D-Biol Crystallogr* **58:** (6 Spec Iss 2) 921.

Clarke NJ, Naylor S*. 2002. *Beyond Genomics Inc, 40 Bear Hill Rd, Waltham, Ma 02451, USA. Capillary isoelectric focusing-mass spectrometry: Analysis of protein mixtures from human body fluids. *Biomed Chromatogr* **16:** (4) 287.

Colantuoni C, Henry G, Zeger S, Pevsner J*. 2002. *Kennedy Krieger Inst, Dept Neurol, 707 Nth Broadway, Baltimore, Md 21205, USA. Local mean normalization of microarray element signal intensities across an array surface: Quality control and correction of spatially systematic artifacts. *Biotechniques* **32:** (6) 1316.

Dorris DR, Ramakrishnan R, Trakas D, Dudzik F, Belval R, Zhao C, Nguyen A, Domanus M, Mazumder A. 2002. Motorola Life Sci, Northbrook, Il 60062, USA. A highly reproducible, linear, and automated sample preparation method for DNA microarrays. *Genome Res* **12:** (6) 976.

Ekstrom S, Malmstrom J, Wallman L, Lofgren M, Nilsson J, Laurell T, Marko-Varga G*. 2002. *AstraZeneca R&D, POB 34, SE-22187 Lund, Sweden. On-chip microextraction for proteomic sample preparation of in-gel digests. *Proteomics* **2:** (4) 413.

Felske A. 2002. GBF, Div Microbiol, Mascheroder Weg 1, DE-38124 Braunschweig, Germany. Streamlined representational difference analysis for comprehensive studies of numerous genomes. *J Microbiol Methods* **50:** (3) 305.

Galeva N, Altermann M*. 2002. *Univ Kansas, Dept Chem, Biochem Res Serv Lab, 1251 Wescoe Hall Dr, Lawrence, Ks 66045, USA. Comparison of one-dimensional and two-dimensional gel electrophoresis as a separation tool for proteomic analysis of rat liver microsomes: Cytochromes P450 and other membrane proteins. *Proteomics* **2:** (6) 713.

Gobry V, Van Oostrum J, Martinelli M, Rohner TC, Reymond F, Rossier JS, Girault HH*. 2002. *Ecole Polytech Fed Lausanne, Lab Electrochim, CH-1015 Lausanne, Switzerland. Microfabricated polymer injector for direct mass spectrometry coupling. *Proteomics* **2:** (4) 405.

Guttman A, Csapo Z, Robbins D. 2002. Torrey Mesa Res Inst, San Diego, Ca 92121, USA. Rapid two-dimensional analysis of proteins by ultra-thin layer gel electrophoresis. *Proteomics* **2:** (4) 469.

Hayes A, Zhang NS, Wu J, Butler PR, Hauser NC, Hoheisel JD, Lim FL, Sharrocks AD, Oliver SG*. 2002. *Univ Manchester, Sch Biol Sci, 2.205 Stopford Bldg, Oxford Rd, Manchester M13 9PT, England. Hybridization array technology coupled with chemostat culture: Tools to interrogate gene expression in *Saccharomyces cerevisiae*. *Methods* **26:** (3) 281.

Jobs M, Howell WM, Brookes AJ*. 2002. *Karolinska Inst, Ctr Genomics & Bioinformatics, Berzelius Vag, SE-17177 Stockholm, Sweden. Creating arrays by centrifugation. *Biotechniques* **32:** (6) 1322.

Keesee SK, Domanik R, Patterson B. 2002. Mol Diagnost Inc, Mol Res, 414 Nth Orleans, Suite 510, Chicago, Il 60610, USA. Fully automated proteomic detection of cervical dysplasia. *Anal Quant Cytol Histol* **24:** (3) 137.

Laoudj-Chenivesse D, Marin P, Bennes R, Tronel-Peyroz E, Leterrier F. 2002. Inst Genet Humaine, CNRS, Assoc Francaise Myopathies, 141 rue Cardonille, FR-34396 Montpellier, France. High performance two-dimensional gel electrophoresis using a wetting agent Tergitol® NP7. *Proteomics* **2:** (5) 481.

Lin HJ, Charles PT, Andreadis JD, Churilla AM, Stenger DA, Pancrazio JJ*. 2002. *US Navy, Ctr Biomol Sci & Engn, 4555 Overlook Ave, Washington, DC 20375, USA. Cholera toxin-induced modulation of gene expression: Elucidation *via* cDNA microarray for rational cell-based sensor design. *Anal Chim Acta* **457:** (1) 97.

Martin K, Hart C, Schulenberg B, Jones L, Patton WF*. 2002. *Mol Probes Inc, Proteomics Sect, 4849 Pitchford Ave, Eugene, Or 97402, USA. Simultaneous reg/green dual fluorescence detection on electroblots using BODIPY TR-X succinimidyl ester and ELF 39 phosphate. *Proteomics* **2:** (5) 499.

Muller PY, Janovjak H, Miserez AR, Dobbie Z. 2002. Univ Basel, Inst Biochem & Genet, Vesalgasse 1, CH-4051 Basel, Switzerland. Pro-

Copyright © 2002 John Wiley & Sons, Ltd.

cessing of gene expression data generated by quantitative real-time RT-PCR. *Biotechniques* **32:** (6) 1372.

Nedelkov D, Tubbs KA, Nelson RW. 2002. Intrinsic Bioprobes Inc, 625 Sth Smith Rd, Tempe, Az 85281, USA. Design of buffer exchange surfaces and sensor chips for biosensor chip mass spectrometry. *Proteomics* **2:** (4) 441.

Oliphant A, Barker DL, Stuelpnagel JR, Chee MS. 2002. Illumina, 9885 Towne Ctr Dr, San Diego, Ca 92121, USA. BeadArray™ technology: Enabling an accurate, cost-effective approach to high throughput genotyping. *Biotechniques* **32:** (Suppl) 56.

Pawlak M, Schick E, Bopp MA, Schneider MJ, Oroszlan P, Ehrat M. 2002. Zeptosens AG, Benkenstr 254, CH-4108 Witterswil, Switzerland. Zeptosens' protein microarrays: A novel high performance microarray platform for low abundance protein analysis. *Proteomics* **2:** (4) 383.

Peterson DG, Schulze SR, Sciara EB, Lee SA, Bowers JE, Nagel A, Jiang N, Tibbitts DC, Wessler SR, Paterson AH. 2002. Univ Georgia, Ctr Appl Genet Technol, Athens, Ga 30602, USA. Integration of Cot analysis, DNA cloning, and high-throughput sequencing facilitates genome characterization and gene discovery. *Genome Res* **12:** (5) 795.

Relogio A, Schwager C, Richter A, Ansorge W, Valcarcel J*. 2002. *EMBL, Gene Express Programme, Meyerhofstr 1, DE-69117 Heidelberg, Germany. Optimization of oligonucleotide-based DNA microarrays (Online paper). *Nucleic Acids Res* **30:** (11) E51.

Shearstone JR, Allaire NE, Getman ME, Perrin S. 2002. Biogen Inc, Transcriptional Profiling Grp, Cambridge Ctr 14, Cambridge, Ma 02142, USA. Nondestructive quality control for microarray production. *Biotechniques* **32:** (5) 1051.

Smith RD, Anderson GA, Lipton MS, Pasa-Tolic L, Shen YF, Conrads TP, Veenstra TD, Udseth HR. 2002. Pacific NW Natl Labs, Environm & Mol Sci Lab, MSIN, K8-98, POB 999, Richland, Wa 99352, USA. An accurate mass tag strategy for quantitative and high-throughput proteome measurements. *Proteomics* **2:** (5) 513.

Spandidos A, Rabbitts TH*. 2002. *MRC Mol Biol Lab, Hills Rd, Cambridge CB2 2QH, England. Sub-proteome differential display: Single gel comparison by 2D electrophoresis and mass spectrometry. *J Mol Biol* **318:** (1) 21.

Tabuchi M, Baba Y. 2002. Univ Tokushima, Dept Med Chem, Tokushima 770 8505, Japan. A novel injection method for high-speed proteome analysis by capillary electrophoresis. *Electrophoresis* **23:** (7-8) 1138.

Tabuchi M, Hino M, Shinohara Y, Baba Y. 2002. Address as above. Cell-free protein synthesis on a microchip. *Proteomics* **2:** (4) 430.

Tahi F, Achddou B, Decraene C, Alibert O, Guiot H, Auffray C, Pietu G. 2002. CNRS Univ Evry Val Essonne, Genopole, Lab LaMI-UMR 8042, 523 Pl Terrasses, FR-91000 Evry, France. Automatic quantitation of hybridization signals on cDNA arrays. *Biotechniques* **32:** (6) 1386.

Tolbert TJ, Wong CH*. 2002. *Scripps Clin & Res Inst, Dept Chem, 10550 Nth Torrey Pines Rd, La Jolla, Ca 92037, USA. New methods for proteomic research: Preparation of proteins with N-terminal cysteines for labeling and conjugation. *Angew Chem Int Ed* **41:** (12) 2171.

Tran PH, Peiffer DA, Shin Y, Meek LM, Brody JP, Cho KWY*. 2002. *Univ Calif Irvine, Dept Dev & Cell Biol, Irvine, Ca 92697, USA. Microarray optimizations: Increasing spot accuracy and automated identification of true microarray signals (Electronic paper). *Nucleic Acids Res* **30:** (12) E54.

Twerenbold D, Netuschill A, De Rooij N, Luginbuhl P, Gerber D, Gritti D, Gonin Y, Rossel F, Vuilleumier JL. 2002. Univ Neuchatel, Inst Phys, rue AL Breguet 1, CH-2000 Neuchatel, Switzerland. Detecting single molecules launched from liquid surfaces in a time-of-flight mass spectrometer using ultraviolet and infrared lasers. *Proteomics* **2:** (4) 436.

Wang YK, Ma Z, Quinn DF, Fu EW. 2002. Novartis Pharmaceut Corp, Cent Technol, Discovery Res, 556 Morris Ave, Summit, NJ 07901, USA. Inverse $^{15}$N-metabolic labeling/mass spectrometry for comparative proteomics and rapid identification of protein markers/targets. *Rapid Commun Mass Spectrom* **16:** (14) 1389.

Wang YM, Wang XJ, Guo SW, Ghosh S. 2002. Children's Hosp, Med Coll Wisconsin, Max McGee Natl Res Ctr Juvenile Diabet, Milwaukee, Wi 53226, USA. Conditions to ensure competitive hybridization in two-color microarray: A theoretical and experimental analysis. *Biotechniques* **32:** (6) 1342.

Weil MR, Macatee T, Garner HR*. 2002. *5323 Harry Hines, Dallas, Tx 75390, USA. Toward a universal standard: Comparing two methods for standardizing spotted microarray data. *Biotechniques* **32:** (6) 1310.

Wierenga SK, Zocher MJ, Mirus MM, Conrads TP, Goshe MB, Veenstra TD*. 2002. *Natl Canc Inst Frederick, SAIC Frederick, Frederick, Md 21702, USA. A method to evaluate tryptic digestion efficiency for high-throughput proteome analyses (Letter). *Rapid Commun Mass Spectrom* **16:** (14) 1404.

Xu CF, Song HW, Yang PY*, Wang Q, Wang HH, Xu YM. 2002. *Fudan Univ, Dept Chem, CN-200433 Shanghai, Peoples Rep China. Modification on the interface of capillary isoelectric focusing and mass spectrometry for proteomic research. *Chem J Chinese Univ-Chinese* **23:** (6) 1035.

Yu JD, Othman MI, Farjo R, Zareparsi S, MacNee SP, Yoshida S, Swaroop A*. 2002. *Univ Michigan, WK Kellogg Eye Ctr, 1000 Wall St, Ann Arbor, Mi 48105, USA. Evaluation and optimization of procedures for target labeling and hybridization of cDNA microarrays. *Mol Vis* **8:** (17) 130.

Yuen T, Wurmbach E, Pfeffer RL, Ebersole BJ, Sealfon SC*. 2002. *CUNY Mt Sinai Sch Med, Department Neurol, New York, NY 10029, USA. Accuracy and calibration of commercial oligonucleotide and custom cDNA microarrays (Online paper). *Nucleic Acids Res* **30:** (10) E48.

Zhou HL, Ranish JA, Watts JD, Aebersold R*. 2002. *Inst Syst Biol, 1441 Nth 34th St, Seattle, Wa 98103, USA. Quantitative proteome analysis by solid-phase isotope tagging and mass spectrometry. *Nat Biotechnol* **20:** (5) 512.

### 16 Bioinformatics

Aguilar D, Oliva B, Aviles FX, Querol E*. 2002. *Univ Autonoma Barcelona, Inst Biotecnol & Biomed, ES-08193 Barcelona, Spain. TranScout: Prediction of gene expression regulatory proteins from their sequences. *Bioinformatics* **18:** (4) 597.

Ahmad S, Gromiha MM. 2002. RIKEN, Tsukuba Inst, 3-1-1 Koyadai, Tsukuba, Ibaraki 305 0074, Japan. NETASA: Neural network based prediction of solvent accessibility. *Bioinformatics* **18:** (6) 819.

Alba MM, Laskowski RA, Hancock JM*. 2002. *Royal Holloway Univ London, Dept Comp Sci, Egham TW20 0EX, England. Detecting cryptically simple protein sequences using the SIMPLE algorithm. *Bioinformatics* **18:** (5) 672.

Babu MM, Sankaran K*. 2002. *Anna Univ, Ctr Biotechnol, IN-600025 Chennai, India. DOLOP: Database of bacterial lipoproteins. *Bioinformatics* **18:** (4) 641.

Berman HM, Westbrook J, Feng ZK, Iype L, Schneider B, Zardecki C *et al*. 2002. Rutgers State Univ, Dept Chem & Chem Biol, 610 Taylor Rd, Piscataway, NJ 08854, USA. The Nucleic Acid Database. *Acta Crystallogr D-Biol Crystallogr* **58:** (6 Spec Iss 1) 889.

Berman HM, Battistuz T, Bhat TN, Bluhm WF, Bourne PE, Burkhardt K, Iype L, Jain S, Fagan P, Marvin J. 2002. Address as above. The Protein Data Bank. *Acta Crystallogr D-Biol Crystallogr* **58:** (6 Spec Iss 1) 899.

Bhalla US. 2002. Natl Ctr Biol Sci, GKVK Campus, IN-560065 Bangalore, India. The chemical organization of signaling interactions. *Bioinformatics* **18:** (6) 855.

Bortoluzzi S, D'Alessi F, Romualdi C, Danieli GA*. 2001. *Univ Padua, Dept Biol, via G Colombo 3, IT-35131 Padua, Italy. Differential expression of genes coding for ribosomal proteins in different human tissues. *Bioinformatics* **17:** (12) 1152.

Bozinov D, Rahnenfuhrer J. 2002. Univ Nebraska, Ctr Human Mol Genet, 600 Sth 42nd St, Omaha, Ne 68198, USA. Unsupervised technique for robust target separation and analysis of DNA microarray spots through adaptive pixel clustering. *Bioinformatics* **18:** (5) 747.

Castelo AT, Martins W, Gao GR. 2002. Univ Delaware, Dept Elect & Comp Engn, 140 Evans Hall, Newark, De 19716, USA. TROLL: Tandem Repeat Occurence Locator. *Bioinformatics* **18:** (4) 634.

Chen ZY, Corey DP. 2002. Massachusetts Gen Hosp, Dept Neurol, Boston, Ma 02114, USA. An inner ear gene expression database. *J Assoc Res Otolaryngol* **3:** (2) 140.

Copyright © 2002 John Wiley & Sons, Ltd.

Colbourn CJ, Ling ACH, Tompa M*. 2002. *Univ Washington, Dept Comp Sci & Engn, Box 352350, Seattle, Wa 98195, USA. Construction of optimal quality control for oligo arrays. *Bioinformatics* **18:** (4) 529.

Coussens PM, Nobis W. 2002. Michigan State Univ, Dept Anim Sci, East Lansing, Mi 48824, USA. Bioinformatics and high throughput approach to create genomic resources for the study of bovine immunobiology. *Vet Immunol Immunopathol* **86:** (3-4) 229.

Del Val C, Ernst P, Brauning R, Glatting KH, Suhai S. 2002. DKFZ, Dept Mol Biophys, INF280, DE-69120 Heidelberg, Germany. PATH: A task for the inference of phylogenies. *Bioinformatics* **18:** (4) 646.

Delcher AL, Phillippy A, Carlton J, Salzberg SL*. 2002. *Inst Genomic Res, 9712 Med Ctr Dr, Rockville, Md 20850, USA. Fast algorithms for large-scale genome alignment and comparison. *Nucleic Acids Res* **30:** (11) 2478.

De Smet F, Mathys J, Marchal K, Thijs G, De Moor B, Moreau Y. 2002. Katholieke Univ Leuven, ESAT-SCD-SISTA, Kasteelpk Arenberg 10, BE-3001 Louvain, Belgium. Adaptive quality-based clustering of gene expression profiles. *Bioinformatics* **18:** (5) 735.

Dutheil J, Galtier N*. 2002. *Univ Montpellier 2, CNRS UMR 5000 Genome Populat Interact, CC 63, Pl E Bataillon, FR-34095 Montpellier, France. BAOBAB: A Java editor for large phylogenetic trees. *Bioinformatics* **18:** (6) 892.

Edgerton ME, Taylor R, Powell JI, Hunter L, Simon R, Liu ET. 2002. Hosp Univ Penn, Dept Pathol & Lab Med, 3400 Spruce St, Philadelphia, Pa 19104, USA. A bioinformatics tool to select sequences for microarray studies of mouse models of oncogenesis. *Bioinformatics* **18:** (5) 774.

Estrada E. 2002. Univ Santiago de Compostela, Dept Organ Chem, Santiago de Compostela, Spain. Characterization of the folding degree of proteins. *Bioinformatics* **18:** (5) 697.

Eyrich VA, Marti-Renom MA, Przybylski D, Madhusudhan MS, Fiser A, Pazos F, Valencia A, Sali A, Rost B*. 2001. *CUBIC Columbia Univ, Dept Biochem & Mol Biophys, 650 West 168th St, New York, NY 10032, USA. EVA: Continuous automatic evaluation of protein structure prediction servers. *Bioinformatics* **17:** (12) 1242.

Fielden MR, Halgren RG, Dere E, Zacharewski TR. 2002. Michigan State Univ, Dept Biochem & Mol Biol, Natl Food Safety & Toxicol Ctr, East Lansing, Mi 48824, USA. GP3: GenePix post-processing program for automated analysis of raw microarray data. *Bioinformatics* **18:** (5) 771.

Fuellen G, Wagele JW, Giegerich R. 2001. Ruhr Univ Bochum, Lehrstuhl Spez Zool, DE-44780 Bochum, Germany. Minimum conflict: A divide-and-conquer approach to phylogeny estimation. *Bioinformatics* **17:** (12) 1168.

Giladi E, Walker MG, Wang JZ, Volkmuth W. 2002. Incyte Pharmaceut, 3174 Porter Dr, Palo Alto, Ca 94304, USA. SST: An algorithm for finding near-exact sequence matches in time proportional to the logarithm of the database size. *Bioinformatics* **18:** (6) 873.

Gonzales MJ, Dugan JM, Shafer RW*. 2002. *Stanford Univ Med Ctr, Div Infect Dis, 300 Pasteur Dr, Room S-156, Stanford, Ca 94305, USA. Synonymous-non-synonymous mutation rates between sequences containing ambiguous nucleotides (Syn-SCAN). *Bioinformatics* **18:** (6) 886.

Gouet P, Courcelle E. 2002. IBCP, CNRS UMR 5086, Lab Biocristallog, 7 Passage Vercors, FR-69367 Lyon 07, France. ENDscript: A workflow to display sequence and structure information. *Bioinformatics* **18:** (5) 767.

Grant JD, Dunbrack RL, Manion FJ, Ochs MF*. 2002. *Fox Chase Canc Ctr, Bioinformatics Working Grp, 7701 Burholme Ave, Philadelphia, Pa 19111, USA. BeoBLAST: Distributed BLAST and PSI-BLAST on a Beowulf cluster. *Bioinformatics* **18:** (5) 765.

Greenbaum D, Jansen R, Gerstein M*. 2002. *Yale University, Department Mol Biophys & Biochem, 266 Whitney Ave, New Haven, Ct 06520, USA. Analysis of mRNA expression and protein abundance data: An approach for the comparison of the enrichment of features in the cellular population of proteins and transcripts. *Bioinformatics* **18:** (4) 585.

Hampson S, Kibler D, Baldi P*. 2002. *Univ Calif, Dept Comp & Informat Sci, Inst Genomics & Bioinformatics, Irvine, Ca 92697, USA. Distribution patterns of over-represented κ-mers in non-coding yeast DNA. *Bioinformatics* **18:** (4) 513.

Helge WA, Bourne PE*. 2002. *Univ Calif San Diego, San Diego Supercomp Ctr, 9500 Gilman Dr, La Jolla, Ca 92093, USA. Protein structure resources. *Acta Crystallogr D-Biol Crystallogr* **58:** (6 Spec Iss 1) 908.

Hoebeke M, Chiapello H, Noirot P, Bessieres P. 2001. INRA CRV, Unite Math Informat & Genome, Route St Cyr, FR-78026 Versailles, France. SPiD: A *subtilis* protein interaction database. *Bioinformatics* **17:** (12) 1209.

Horimoto K, Toh H. 2001. Saga Med Sch, Lab Math, 5-1-1 Nabeshima, Saga 849 8501, Japan. Statistical estimation of cluster boundaries in gene expressions profile data. *Bioinformatics* **17:** (12) 1143.

Hoyle DC, Rattray M, Jupp R, Brass A. 2002. Univ Manchester, Sch Biol Sci, Stopford Bldg, Oxford Rd, Manchester M13 9PT, England. Making sense of microarray data distributions. *Bioinformatics* **18:** (4) 576.

Jolley KA, Feil EJ, Chan MS, Maiden MCJ. 2001. Univ Oxford, Dept Zool, Sth Parks Rd, Oxford OX1 3PS, England. Sequence type analysis and recombinational tests (START). *Bioinformatics* **17:** (12) 1230.

Junier T, Pagni M, Bucher P. 2001. Swiss Inst Bioinformatics, Epalinges, Switzerland. mmsearch: A motif arrangement language and search program. *Bioinformatics* **17:** (12) 1234.

Karreman C. 2002. Univ Dusseldorf, Dept Oncol Chem, Bldg 23-12-U1, Universitatsstr 1, DE-40225 Dusseldorf, Germany. AiO, combining DNA/protein programs and oligo-management. *Bioinformatics* **18:** (6) 884.

Kent WJ, Sugnet CW, Furey TS, Roskin KM, Pringle TH, Zahler AM, Haussler D. 2002. Univ Calif, Dept Mol Cellular & Dev Biol, Santa Cruz, Ca 95064, USA. The human genome browser at UCSC. *Genome Res* **12:** (6) 996.

Kose F, Weckwerth W, Linke T, Fiehn O. 2001. Max-Planck-Inst, Mol Plant Physiol, Dept Lothar Wollmitzer, Postfach, DE-14424 Potsdam, Germany. Visualizing plant metabolomic correlation networks using clique-metabolite matrices. *Bioinformatics* **17:** (12) 1198.

Kumar S, Tamura K, Jakobsen IB, Nei M. 2001. Arizona State Univ, Dept Biol, Tempe, Az 85287, USA. MEGA2: Molecular evolutionary genetics analysis software. *Bioinformatics* **17:** (12) 1244.

Leontovich AM, Brodsky LI, Drachev VA, Nikolaev VK*. 2002. *Moscow MV Lomonsov State Univ, Belozersky Inst Physicochem Biol, RU-119899 Moscow, Russia. Adaptive algorithm of automated annotation. *Bioinformatics* **18:** (6) 838.

Li JY, Wong LS. 2002. Kent Ridge Digital Labs, 21 Heng Mui Keng Terrace, SG-119613 Singapore, Rep Singapore. Identifying good diagnostic gene groups from gene expression profiles using the concept of emerging patterns. *Bioinformatics* **18:** (5) 725.

Li LP, Weinberg CR, Darden TA, Pedersen LG. 2001. NIEHS, Biostat Branch, Res Triangle Park, NC 27709, USA. Gene selection for sample classification based on gene expression data: Study of sensitivity to choice of parameters of the GA/KNN method. *Bioinformatics* **17:** (12) 1131.

Liang J, Kachalo S. 2002. Univ Illinois, Dept Bioengn, 851 Sth Morgan St, Chicago, Il 60607, USA. Computational analysis of microarray gene expression profiles: Clustering, classification, and beyond. *Chemometr Intell Lab Syst* **62:** (2) 199.

Lombard V, Camon EB, Parkinson HE, Hingamp P, Stoesser G, Redaschi N. 2002. European Bioinformat Inst, EMBL Outstn, Wellcome Trust Genome Campus, Cambridge CB10 1SD, England. EMBL-Align: A new public nucleotide and amino acid multiple sequence alignment database. *Bioinformatics* **18:** (5) 763.

Loots GG, Ovcharenko I, Pachter L, Dubchak I, Rubin EM. 2002. Univ Calif Berkeley, Genome Sci Dept, Berkeley, Ca 94720, USA. rVista for comparative sequence-based discovery of functional transcription factor binding sites. *Genome Res* **12:** (5) 832.

Luyf ACM, De Gast J, Van Kampen AHC*. 2002. *Univ Amsterdam, Acad Med Ctr, Bioinformatics Lab, Meibergdreef 9, NL-1105 AZ Amsterdam, The Netherlands. Visualizing metabolic activity on a genome-wide scale. *Bioinformatics* **18:** (6) 813.

Marc P, Jacq C. 2002. Ecole Normale Super, Lab Mol Genet, UMR CNRS 8541, 46 rue Ulm, FR-75005 Paris, France. Arrayplot for visualization and normalization of cDNA microarray data. *Bioinformatics* **18:** (6) 888.

Martin RG, Rosner JL. 2002. NIH/NIDDK, Mol Biol Lab, Bethesda, Ma 20892, USA. Genomics of the *marA/soxS/rob* regulon of *Escherichia*

Copyright © 2002 John Wiley & Sons, Ltd. 

*coli*: Identification of directly activated promoters by application of molecular genetics and informatics to microarray data. *Mol Microbiol* **44:** (6) 1611.

Moloshok TD, Klevecz RR, Grant JD, Manion FJ, Speier WF, Ochs MF*. 2002. *Fox Chase Canc Ctr, Bioinformatics Working Grp, Philadelphia, Pa 19111, USA. Application of Bayesian decomposition for analysing microarray data. *Bioinformatics* **18:** (4) 566.

Morgenstern B, Rinner O, Abbeddaim S, Haase D, Mayer KFX, Dress AWM, Mewes HW. 2002. Univ Bielefeld, Tech Fak, Postfach 10.01. 31, DE-33501 Bielefeld, Germany. Exon discovery by genomic sequence alignment. *Bioinformatics* **18:** (6) 777.

Ochagavia ME, Richelle J, Wodak SJ*. 2002. *Free Univ Brussels, Serv Conformat Macromol Biol & Bioinformat, Ave F Roosevelt, BE-1050 Brussels, Belgium. Advanced pairwise structure alignments of proteins and analysis of conformational changes. *Bioinformatics* **18:** (4) 637.

Oyama T, Kitano K, Satou K, Ito T. 2002. INTEC Web, 3-23 Shimoshin machi, Toyama 930 0804, Japan. Extraction of knowledge on protein-protein interaction by association rule discovery. *Bioinformatics* **18:** (5) 705.

Paley SM, Karp PD*. 2002. *SRI Int, Bioinformatics Res Grp, 333 Ravenswood Ave, Menlo Park, Ca 94025, USA. Evaluation of computational metabolic-pathway predictions for *Helicobacter pylori*. *Bioinformatics* **18:** (5) 715.

Peleg M, Yeh I, Altman RB*. 2002. *Stanford Univ, Stanford, Ca 94305, USA. Modelling biological processes using workflow and Petri Net models. *Bioinformatics* **18:** (6) 825.

Ponger L, Mouchiroud D. 2002. Univ Lyon 1, Lab Biometrie & Biol Evolut, CNRS UMR 5558, 43 Blvd 11 Novembre 1918, FR-69622 Villeurbanne, France. CpGProD: Identifying CpG islands associated with transcription start sites in large genomic mammalian sequences. *Bioinformatics* **18:** (4) 631.

Raval A, Ghahramani Z, Wild DL*. 2002. *Keck Grad Inst Appl Life Sci, 535 Watson Dr, Claremont, Ca 91711, USA. A Bayesian network model for protein fold and remote homologue recognition. *Bioinformatics* **18:** (6) 788.

Ray WC, Munson RS, Daniels CJ. 2001. Ohio State Univ, Childrens Res Inst, 700 Childrens Dr Columbus, Columbus, Oh 43205, USA. Tricross: Using dot-plots in sequence-id space to detect uncatalogued intergenic features. *Bioinformatics* **17:** (12) 1105.

Reeve JP, Rannala B*. 2002. *University Alberta, Dept Med Genet, 8-39 Med Sci Bldg, Edmonton, Alberta, Canada T6G 2H7. DMLE+: Bayesian linkage disequilibrium gene mapping. *Bioinformatics* **18:** (6) 894.

Rusconi F, Belghazi M. 2002. Univ Bordeaux I, LPTC UMR CNRS 5472, FR-33405 Talence, France. Desktop prediction/analysis of mass spectrometric data in proteomic projects by using *massXpert*. *Bioinformatics* **18:** (4) 644.

Rychlewski L. 2001. BioInfoBank Inst, Bioinformatics Lab, ul Limanowskiego 24A, PL-60744 Poznan, Poland. ToolShop: Prerelease inspections for protein of structure prediction servers. *Bioinformatics* **17:** (12) 1240.

Schlosshauer M, Ohlsson M. 2002. Lund Univ, Dept Theoret Phys, Complex Syst Div, Solvegatan 14A, SE-22362 Lund, Sweden. A novel approach to local reliability of sequence alignments. *Bioinformatics* **18:** (6) 847.

Schreiber M, Brown C*. 2002. *Univ Otago, Dept Biochem, POB 56, Dunedin, New Zealand. Compensation for nucleotide bias in a genome by representation as a discrete channel with noise. *Bioinformatics* **18:** (4) 507.

Shmulevich I, Zhang W. 2002. UT MD Anderson Canc Ctr, Canc Genomics Lab, 1515 Holcombe Blvd, Houston, Tx 77030, USA. Binary analysis and optimization-based normalization of gene expression data. *Bioinformatics* **18:** (4) 555.

Sommer I, Zien A, Von Ohsen N, Zimmer R, Lengauer T. 2002. Fraunhofer Inst Algorithms & Sci Comp, Schloss Birlinghoven, DE-53754 Sankt Augustin, Germany. Confidence measures for protein fold recognition. *Bioinformatics* **18:** (6) 802.

Stanton JL, Macgregor AB, Green DPL*. 2002. *Univ Otago, Dept Anat & Struct Biol, POB 913, Dunedin, New Zealand. Using expressed sequence tag databases to identify ovarian genes of interest. *Mol Cell Endocrinol* **191:** (1) 11.

Suzek BE, Ermolaeva MD, Schreiber M, Salzberg SL*. 2001. *Johns Hopkins Univ, Dept Comp Sci, Baltimore, Md 21218, USA. A probabilistic, method for identifying start codons in bacterial genomes. *Bioinformatics* **17:** (12) 1123.

Thijs G, Lescot M, Marchal K, Rombauts S, De Moor B, Rouze P, Moreau Y. 2001. Katholieke Univ Leuven, ESAT-SISTA/KOSIC, Kasteelpk Arenberg 10, BE-3001 Heverlee, Belgium. A higher-order background model improves the detection of promoter regulatory elements by Gibbs sampling. *Bioinformatics* **17:** (12) 1113.

Togawa RC, Antoniw JF, Mullins JGL*. 2001. *Univ Luton, Fac Sci Technol & Design, Dept Biol & Hlth Sci, Membrane Protein Grp, Park Sq, Luton LU1 3JU, England. TMCompare: Transmembrane region sequence and structure. *Bioinformatics* **17:** (12) 1238.

Troyanskaya OG, Arbell O, Koren Y, Landau GM, Bolshoy A*. 2002. *Univ Haifa, Inst Evolut, Genome Div, IL-31999 Haifa, Israel. Sequence complexity profiles of prokaryotic genomic sequences: A fast algorithm for calculating linguistic complexity. *Bioinformatics* **18:** (5) 679.

Von Mering C, Krause R, Snel B, Cornell M, Oliver SG, Fields S, Bork P*. 2002. *EMBL, Meyerhofstr 1, DE-69012 Heidelberg, Germany. Comparative assessment of large-scale data sets of protein-protein interactions. *Nature* **417:** (6887) 399.

Wagner A. 2001. Univ New Mexico, 167A Castetter Hall, Albuquerque, NM, USA. How to reconstruct a large genetic network from $n$ gene perturbations in fewer than $n^2$ easy steps. *Bioinformatics* **17:** (12) 1183.

Webber C, Barton GJ*. 2001. *Univ Dundee, Sch Life Sci, Dow St, Dundee DD1 5EH, Scotland. Estimation of P-values for global alignments of protein sequences. *Bioinformatics* **17:** (12) 1158.

Wildgruber R, Reil G, Drews O, Parlar H, Gorg A*. 2002. *Tech Univ Munich, Fachgebiet Proteomik, Forum 2, DE-85350 Freising Weihenstephan, Germany. Web-based two-dimensional database of *Saccharomyces cerevisiae* proteins using immobilized pH gradients from pH 6 to pH 12 and matrix-assisted laser desorption/ionization-time of flight mass spectrometry. *Proteomics* **2:** (6) 727.

Wildpaner M, Schneider G, Schleiffer A, Eisenhaber F*. 2001. *Res Inst Mol Pathol, Dr Bohr Gasse 7, AU-1030 Vienna, Austria. Taxonomy workbench. *Bioinformatics* **17:** (12) 1179.

Wren JD, Mittelman DA, Garner HR. 2002. UT SW Med Ctr, Biomed Sci, 5323 Harry Hines Blvd, Dallas, Tx 75390, USA. SIGNAL: Sequence Information and GeNomic AnaLysis. *Comput Methods Program Biomed* **68:** (2) 177.

Wruck W, Griffiths H, Steinfath M, Lehrach H, Radelof U, O'Brien J. 2002. Max-Planck-Inst Mol Genet, Ihnestr 73, DE-14195 Berlin, Germany. Xdigitise: Visualization of hybridization experiments. *Bioinformatics* **18:** (5) 757.

Xu Y, Olman V, Xu D. 2002. Oak Ridge Natl Lab, Protein Informatics Grp, MS 6480, Oak Ridge, Tn 37831, USA. Clustering gene expression data using a graph-theoretic approach: An application of minimum spanning trees. *Bioinformatics* **18:** (4) 536.

Xu Y, Selaru FM, Yin J, Zou TT, Shustova V, Mori Y, Sato F, Liu TC, Olaru A, Wang S *et al*. 2002. c/o Meltzer SJ, Univ Maryland, Rm N3W65, 22 Sth Greene St, Baltimore, Md 21201, USA. Artificial neural networks and gene filtering distinguish between global gene expression profiles of Barrett's esophagus and esophageal cancer. *Cancer Res* **62:** (12) 3493.

Yu YK, Bundschuh R, Hwa T. 2002. Florida Atlantic University, Department Phys, 777 Glades Rd, Boca Raton, Fl 33431, USA. Hybrid alignment: High-performance with universal statistics. *Bioinformatics* **18:** (6) 864.

Zavaljevski N, Stevens FJ, Reifman J*. 2002. *US Army, Med Res & Mat Command, 504 Scott St, Fort Detrick, Md 21702, USA. Support vector machines with selective kernel scaling for protein classification and identification of key amino acid positions. *Bioinformatics* **18:** (5) 689.

Zhang T, Zhang M*. 2001. *Cold Spring Harbor Lab, POB 100, Cold Spring Harbor, NY 11724, USA. Promoter Extraction from GenBank (PEG): Automatic extraction of eukaryotic promoter sequences in large sets of genes. *Bioinformatics* **17:** (12) 1232.

Zhu W, Brendel V. 2002. Iowa State Univ, Dept Zool & Genet, Ames, Ia 50011, USA. Gene structure identification with MyGV using cDNA evidence and protein homologs to improve *ab initio* predictions. *Bioinformatics* **18:** (5) 761.

Copyright © 2002 John Wiley & Sons, Ltd. 
